# Impact of Marine Chemical Ecology Research on the Discovery and Development of New Pharmaceuticals

**DOI:** 10.3390/md21030174

**Published:** 2023-03-09

**Authors:** Lik Tong Tan

**Affiliations:** Natural Sciences and Science Education, National Institute of Education, Nanyang Technological University, Singapore 637616, Singapore; liktong.tan@nie.edu.sg

**Keywords:** marine chemical ecology, allelochemicals, infochemicals, specialized metabolites, drug discovery, marine symbioses, chemical defense, climate change

## Abstract

Diverse ecologically important metabolites, such as allelochemicals, infochemicals and volatile organic chemicals, are involved in marine organismal interactions. Chemically mediated interactions between intra- and interspecific organisms can have a significant impact on community organization, population structure and ecosystem functioning. Advances in analytical techniques, microscopy and genomics are providing insights on the chemistry and functional roles of the metabolites involved in such interactions. This review highlights the targeted translational value of several marine chemical ecology-driven research studies and their impact on the sustainable discovery of novel therapeutic agents. These chemical ecology-based approaches include activated defense, allelochemicals arising from organismal interactions, spatio-temporal variations of allelochemicals and phylogeny-based approaches. In addition, innovative analytical techniques used in the mapping of surface metabolites as well as in metabolite translocation within marine holobionts are summarized. Chemical information related to the maintenance of the marine symbioses and biosyntheses of specialized compounds can be harnessed for biomedical applications, particularly in microbial fermentation and compound production. Furthermore, the impact of climate change on the chemical ecology of marine organisms—especially on the production, functionality and perception of allelochemicals—and its implications on drug discovery efforts will be presented.

## 1. Introduction

Marine chemical ecology is a relatively young science and focuses on the study of chemically mediated interactions between marine organisms. Such study can provide insights into the ecology and evolution of marine populations and communities and the function of marine ecosystems [1]. The search for novel marine-derived compounds has largely been driven by the need to discover potentially useful compounds, and studies on the ecological importance of these molecules has unfortunately been lagging. However, since its early beginnings in the 1980′s, numerous marine natural products have been investigated for their ecological functions. A wide range of chemically mediated interactions have been explored, including predator–prey and seaweed–herbivore interactions, chemical defenses against fouling organisms and pathogenic marine microbes, competitive interactions, settlement cues, and interactions between planktonic organisms [1,2,3,4]. Currently, there is a great research interest in the nature of chemical signals or infochemicals regulating the host–microbial interactions of marine holobionts as well as marine planktons, which form the basis of the marine food web and have a significant influence on carbon and nutrient cycles [5,6]. Such chemical ecology research is driven by rapid advances in omic technologies, including metabolomics and genomics, for uncovering the structures and functions of signaling molecules [7].

The information from the ecological and evolutionary studies of these marine-derived molecules, particularly pertaining to how, why, where and when they are produced, can be useful in drug discovery efforts. Instead of the random screening of marine organisms, chemical ecology-driven approaches can lead to the sustainable discovery of structurally novel bioactive compounds and new sources of analogues or identical molecules and improve the production of bioactive compounds. This is because many of the challenges that need to be solved by new technologies, including new medicines and materials, are also issues that are faced by marine organisms [8]. Thus, investigations of appropriate natural ecology-based models could provide a more direct access to the discovery of useful molecules. It is also important to recognize that the bioprospecting of useful molecules from marine organisms is a result of evolution by natural selection to bind to specific receptors in ecological targets. For instance, allelochemicals used in competition or defense by benthic marine organisms such as sponges and soft corals are sources of potent cytotoxic molecules as they ward off competitors by causing apoptosis, autophagy or necrosis [9]. In addition, in preventing the settlement of larvae and in reducing cell damage caused by harmful ultraviolet (UV) rays, benthic invertebrates produce antifouling and UV-protective compounds, respectively. These molecules could potentially be used in the development of novel antifouling technologies and UV sunscreens [10].

Unraveling the ecological functions of certain marine-based systems/interactions have implications for human health, particularly for the development of new therapeutics and novel molecular targets. For instance, NF-kB (nuclear factor kappa light chain enhancer of activated B cells) is an ancient protein complex that is involved in the regulation of immune response, cell growth and survival, as well as development. Interestingly, NF-kB and related systems are present in marine organisms, including marine invertebrates (e.g., sea hares, sea urchins, ascidians, crustaceans and mussels) and fish. NF-kB and related systems in marine organisms are activated by biotic and abiotic factors, such as UV radiation, oxidative stress/hypoxia, salinity/pH acclimatization, parasitism and symbiotic interactions. As such, it is not surprising that NF-kB inhibitors are found in marine organisms to protect and defend organisms against UV radiation, oxidative stress and parasites by downregulating the activation of NF-kB [11]. Since NF-kB is also implicated in diseases such as cancer and inflammation, the production of marine-based NF-kB inhibitors are potential drug candidates [12]. Recently, a coral-associated bacterium, New 33, was found to inhibit NF-kB via a non-canonical pathway without causing cytotoxicity [13]. Since the NF-kB system is also found in cnidarians, this finding supports the involvement of the protein complex in host–microbe symbiosis and has therapeutic applications in the modulation of mammalian NF-kB associated with cancer diseases, arthritis and asthma. In addition, chemical ecology studies of eukaryotic–prokaryotic or prokaryotic–prokaryotic interactions could provide insights into possible novel molecular targets of ecologically active compounds relevant to treating human ailments such as microbial infectious. Furthermore, chemical ecology investigations of host–symbiont symbioses, particularly in marine holobionts, can provide an understanding of the roles of microflora/microbiome in human health [14].

Apart from human health, infochemical-based communication has potential biotechnological applications, particularly in aquaculture, marine conservation, management and biodiversity restoration (refer to reviews in references [15,16]). The key benefit of infochemicals is the efficient transmittance of communication across taxonomic groups, kingdoms of life and different trophic levels, ranging from interactions between the simplest unicellular organisms to complex multicellular plants and animals. For example, the hydrophobic toxins released in the algal lysed coral polyps of the branching coral *Acropora nasuta* attracts the herbivorous gobies to feed on the algal fronds of the *Chlorodesmis* sp. [17]. The gobies in turn become more toxic to predators, which could drive a further symbiotic relationship with *A. nasuta*. Deciphering the intricate functions of chemical cues used in complex organismal interactions can provide potential novel strategies to enhance the blue economy. The preliminary evidence of such strategies has been demonstrated in several areas, including pest control in fisheries, biofouling prevention and reef rehabilitation. The use of the turbot-derived compound 2-aminoacetophenone to prevent infection in farmed salmon by the parasitic sea lice [18], formulation of environmentally friendly antifouling coatings based on macroalgae and sponge extracts and on submerged structures [19], and the promoting of coral larval settlement induced by chemical cues from crustose coralline algae in reef rehabilitation efforts [20] are some examples of such strategies.

This review presents the value of marine chemical ecology research and its implications for the discovery and development of novel marine-derived bioactive compounds with pharmaceutical potentials. These compounds play important ecological roles in various organismal interactions and have applications in drug discovery efforts. In addition, a brief overview of innovative analytical and genomic techniques used for the detection of ecologically active specialized molecules is discussed. In view of challenges faced by climate change, the effects of abiotic factors, including the increased surface water temperature and CO_2_ concentration, on the functionality and production of marine natural products will also be presented.

## 2. Chemical Ecology-Driven Discovery of Marine Medicines

### 2.1. Marine Macrobiota–Microbial Interactions

In the marine environment, macroorganisms, such as macroalgae, sponges and corals, are constantly threatened by antagonistic encounters with pathogens, predators and parasites. These interactions exert strong selective pressures on the evolution of defensive strategies, including chemical defense and immune responses in the macroorganisms. The production of these defensive molecules can either be derived from the macroorganisms or their microbial symbionts. As such, ecological studies on the diverse chemicals arising from host–microbial interactions could lead to the discovery of potent novel medicines.

#### 2.1.1. Macroalgal Chemical Defenses against Microbial Attacks

**Figure 1:**
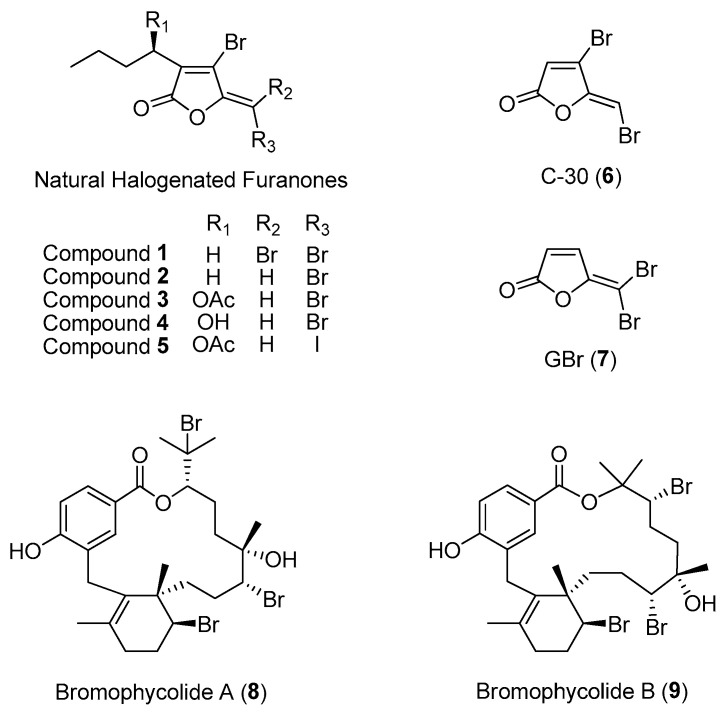
Specialized metabolites **1**–**9** involved in macroalgae chemical defenses against microbial attacks.

One of the best-documented studies on marine macroalgal chemical defenses is the marine red alga, *Delisea pulchra*. *D. pulchra* is a dominant component of the seaweed community and is found predominantly in both temperate and subtropical Australia. Local herbivores tend to avoid preying on this alga, and their thallus are usually found to be unfouled. To date, at least 20 derivatives of halogenated furanones, with a variety of associated ecological functions, have been identified from this marine red algal species [21]. These compounds are enclosed in specialized algal gland cells, which are located within and on the surface of the thallus [22]. The halogenated furanones, e.g., compounds **1** to **5** (Figure 1), possess a broad range of biological activities, such as preventing local herbivores and eukaryotic fouling organisms and warding off marine microbial pathogens. Moreover, the furanones were found to protect algal sporelings from bleaching by preventing opportunistic bacterial infections in the seaweed [21].

The mechanism of action of algal halogenated furanones is a classic example of how marine macroorganisms can inhibit prokaryotic communication signals. Gram-negative bacteria are known to use quorum-sensing chemical signals such as acylated homoserine lactones (AHLs) to control the expression of many bacterial traits, including surface colonization, biofilm formation and pathogenicity [23]. These acylated homoserine lactones bind to bacterial transcription protein to initiate the expression of bacterial traits. It turns out that algal halogenated furanones interfere with the bacterial communication system by binding to the same receptor binding sites of acylated homoserine lactones, resulting in the inhibition of AHL-mediated quorum sensing among bacteria [24]. Hence, the production of halogenated furanones represents an effective defense strategy used by the seaweed in preventing bacterial infection [21]. It was also discovered that natural halogenated furanones and their synthetic analogs, e.g., C-30 (**6**) and GBr (**7**) (Figure 1), inhibit quorum sensing in *Pseudomonas aeruginosa*, highlighting their potential application as anti-infective agents against pathogenic bacteria [25,26].

Advances in analytical chemistry techniques can facilitate the identification of bioactive compounds that are involved in eukaryotic–microbe interactions on native surfaces. For instance, Lane and co-workers were the first to employ an imaging mass spectrometry technique, such as desorption electrospray ionization mass spectrometry, to detect and quantify a series of antifungal algal molecules (bromophycolides) on the surface of the tropical red alga, *Callophycus serratus* [27]. This analytical technique revealed an unequal distribution of bromophycolides A (**8**) and B (**9**) (Figure 1) on the surface of the algal thallus. The natural concentrations of these two molecules were also found to be more than sufficient to prevent the growth of the marine fungal pathogen *Lindra thalassiae*, which known to infect other marine plants, including brown algae and seagrasses. This study illustrates that integrating analytical technique with the high spatial resolution analysis of a bacterial community would be a powerful tool to determine the impact of surface chemistry on the host-associated microbial diversity [28]. Of the bromophycolide series, bromophycolide A is currently being explored as a potential antimalarial and antiviral agent [29,30].

#### 2.1.2. Chemical Defenses Mediated by Marine Invertebrate-Associated Microbial Symbionts

**Figure 2:**
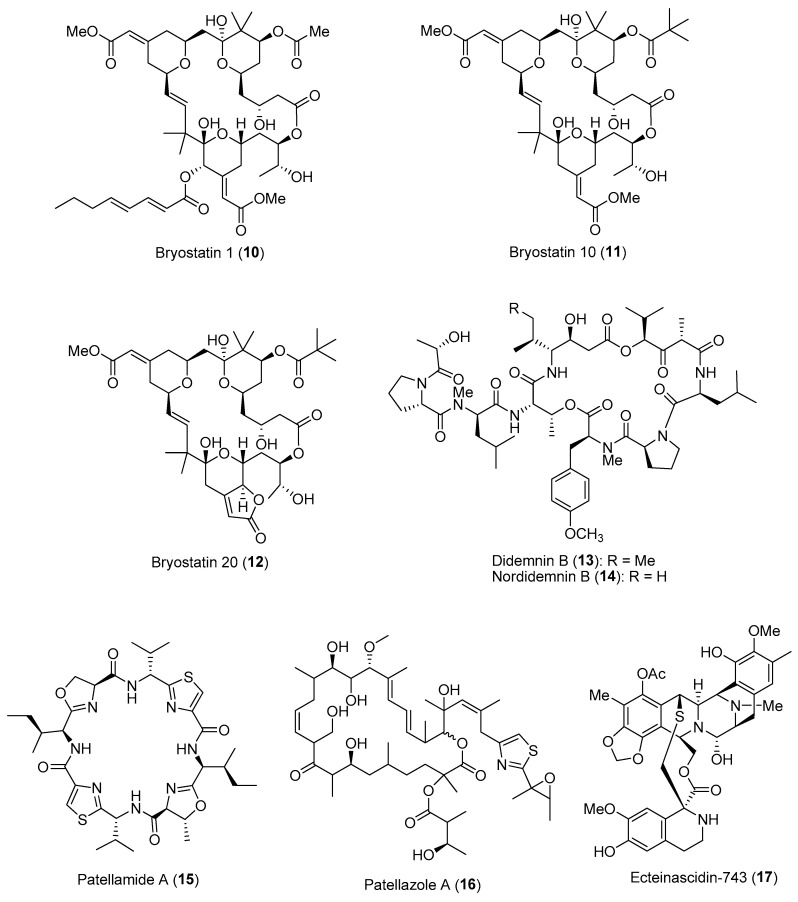
Specialized metabolites involved in chemical defenses mediated by marine invertebrate-associated microbial symbionts.

Several pharmaceutically important compounds originally reported from marine animals, such as sponges, bryozoans and tunicates, have been conclusively shown to be produced by their microbial symbionts [31]. These microbial-derived compounds confer important ecological functions, such as chemical defense and antifouling activities, to the host organisms. Revealing the ecological roles of these symbiotic microbial molecules would not only expediate the discovery of novel bioactive compounds, but also address the supply issues of compounds for downstream drug development. One such example is the discovery of the anticancer macrocyclic lactone, bryostatin 1 (**10**) (Figure 2), from the temperate marine bryozoan *Bugula neritina*. To date, about 20 different members of the bryostatin class of molecules have been reported, with bryostatin 1 currently in clinical trial for the treatment of Alzheimer’s disease [32]. Since the initial report of bryostatin 1 from *B. neritina*, it was hypothesized that the true biosynthetic source of this molecule originated from symbiotic microbes. Previous studies have reported on the consistent association of rod-shaped bacteria with adult and larval *B. neritina*, which were subsequently named ‘*Candidatus* Endobugula sertula’, a γ-Proteobacterium. Further investigations based on chemical biology techniques have provided evidence that the bryostatins are indeed produced by the symbiotic γ-Proteobacterial strain [33]. These compounds, particularly bryostatins 10 (**11**) and 20 (**12**) (Figure 2), are usually found in relatively lower concentrations in adult *B. neritina* colonies as compared to their larval form, providing chemical protection from predation [34]. The bryostatins’ toxicity and deterrence to predators such as fishes could be due to their specific binding to the regulatory domain of protein kinase C and its activation in the mammalian system. Interestingly, the symbiont-produced bryostatins were found to be important cues for reproduction in the host animal via activation of protein kinase C [35].

An amazing array of bioactive natural products ranging from alkaloids to cyclic peptides have been discovered from marine tunicates. It has been predicted that about 8% of the reported secondary metabolites from tunicates (>1000 compounds) are produced by symbiotic microbes [36]. Due to the highly toxic nature of these compounds, they provide the host animal with chemical protection from predation, along with other ecological functions, including metal ion sequestration. The colonial ascidians of the family Didemnidae have been investigated extensively as sources of important bioactive compounds such as cyclic peptides and didemnins, which were originally reported from the Caribbean ascidian, *Trididemnum solidum*. This animal is also host to the cyanobacterial symbiont *Synechocystis trididemni*, which is possibly the true producer of the cyclic peptides. This is due to certain structural motifs of didemnins, such as *N*,*O*-dimethyl tyrosine and two ketide-extended amino acid units [isostatine and 2-(2-hydroxyisovaleryl)propionate], consistent with the diagnostic features of cyanobacterial metabolism. Moreover, at least two α-Proteobacterial strains of *Tistrella mobilis,* YIT 12409 and KA081020-06, have been reported to produce didemnins [37,38]. It has been speculated that the cyanobacterial symbiont acquired the potential for didemnin biosynthesis via horizontal gene transfer, possibly from an α-Proteobacterial strain. Investigation of the ecological roles of didemnins revealed that didemnin B (**13**) and nordidemnin B (**14**) (Figure 2) significantly prevented predation by coral reef fishes when the compounds were added into palatable feeding pellets at concentrations below their levels in adult *T. solidum* colonies [39]. In addition, these cyclic depsipeptides induced regurgitation in allopatric fish predators, and these predators later learned aversion to these compounds [40]. Moreover, both didemnin B and nordidemnin B were present on *T. solidum* larvae at concentrations that could account for their rejection by predatory foraging fishes.

Extensive biochemical research on another large class of ribosomal cyclic peptides, the cyanobactins, produced by the cyanobacterial symbiont *Prochloron didemni* of the tunicate host *Lissoclinum patella* provided insights into the chemical-diversity-driven capacity of the microbes [41]. These cyanobactins include the highly cytotoxic patellamides [e.g., patellamide A (**15**)], trunkamide, lissoclinamides and patellins (Figure 2). Such biochemical insights can be applied to the rationally engineered creation of large libraries of synthetic compounds for lead compound identification [42]. The ecological roles of these symbiont-derived cyanobactins have been proposed, including metal ion sequestration and/or transport, chemical defense and catalysis and/or transport of substrates [43]. Along with *P. didemni*, the tunicate host also houses a novel symbiotic α-proteobacterium, Ca. Endolissoclinum faulkneri, which produces the highly toxic polyketides—patellazoles, e.g., patellazole A (**16**) (Figure 2) [44]. Based on metagenome sequencing and assembly, it was revealed that the bacterial genome was in an advanced state of degradation, where the patellazole genes (about 65 kbp) comprised approximately 10% of the coding sequence of the microbe. This reflects the essential role of the molecule in symbiosis, possibly conferring chemical defense to the host animal. Similarly, a γ-Proteobacterium, Ca. Endoecteinascidia frumentensis, was revealed via meta-omics approaches to be the producer of ecteinascidin-743 (**17**) (Figure 2), an approved anticancer drug. This bacterium is a symbiont of the host tunicate *Ecteinascidia turbinata*, and the molecule is hypothesized to confer protection to the tunicate larvae from fish predation. Moreover, the dispersion of ecteinascidin-743 biosynthetic genes detected throughout the highly reduced genome (about 631 kbp) indicates that production of the molecule is most likely essential to the microbial–host interaction [44].

**Figure 3:**
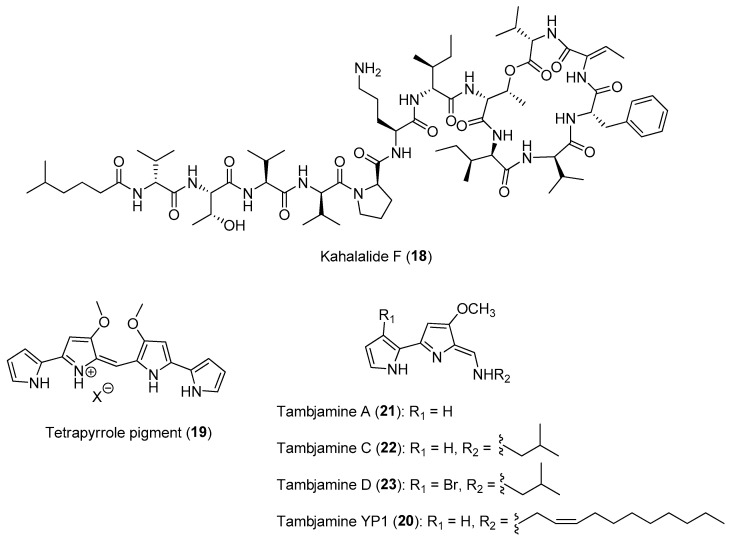
Specialized metabolites involved in chemical defenses mediated by marine invertebrate-associated microbial symbionts.

A recent investigation into the biogenetic source of a class of potent anticancer compounds, the kahalalides, e.g., kahalalide F (**18**) (Figure 3), led to the discovery of a novel obligate marine bacterium, *Candidatus* Endobryopsis kahalalidefaciens, as the producer of the molecules. The marine bacterium is found living intracellularly in the marine algal host, *Bryopsis* sp. [45]. The defensive kahalalides confer chemical protection to the *Bryopsis* sp. as well as to its predatory sacoglossan mollusk, *Elysia rufescens*, which obtains the kahalalides through its algal diet [46]. Certain species of sacoglossans are known to consume algae and digest the algal cells but maintaining functional chloroplasts. A recent study by Torres and co-workers provided experimental evidence of the involvement of sequestered chloroplasts in the production of defensive polypropionates in certain mollusk species, such as *E. chlorotica* and *E. timida* [4,47]. Interestingly, from metatranscriptomic analysis, about 26% of the transcriptional activity of the obligate marine bacterium Ca. Endobryopsis kahalalidefaciens is related to kahalalide biosynthesis, with varying expression levels of different kahalalides. This revelation highlights the importance of these molecules in maintaining a successful agal–bacterium symbiosis. Such tripartite interaction involving a bacterium, an alga and a predatory animal is probably widespread in marine ecosystem and highlights the evolution of complex symbiotic relationships related to chemical defense.

Another example of tripartite interaction is the sequestration of the bispyrrole alkaloids known as tambjamines by several nudibranch species from sessile marine invertebrates, such as bryozoans and tunicates. These alkaloids are possibly produced by symbiotic microbes associated with sessile invertebrate hosts. Evidence for the microbial symbionts’ source of the alkaloids is based on the isolation of related tetrapyrrole pigment (**19**) and a tambjamine analog, tambjamine YP1 (**20**), from the microbes, *Serratia marcescens* and *Pseudoalteromonas tunicata*, respectively (Figure 3) [48,49]. These microbial-derived tambjamines are hypothesized to provide chemical defenses to both invertebrate hosts and mobile invertebrates that obtain these metabolites through their diet. In nature, the carnivorous nudibranch *Roboastra tigris* feeds preferentially on two other nudibranchs, *Tambja abdere* and *T. eliora*. Ecological studies revealed that when the prey *T. abdere* was attacked by *R. tigris*, it secretes a distasteful mucus containing high concentrations of tambjamines that would cause *R. tigris* to break off the attack [50]. In addition, the presence of these alkaloids in the invertebrate hosts, such as bryozoans and tunicates, could prevent marine protozoan grazing, as tambjamine YP1 has been found to cause the fast killing of the nematode *Caenorhabditis elegans* [51,52]. Furthermore, studies of the chemical defenses of the Indo-Pacific tunicate *Atapozoa* sp. (=*Sigillina signifera*) showed that several tambjamines found in the tissues of the ascidian obtained from different locations prevented predation by generalist fishes in field assays [53]. Recent metabolomic analysis revealed that the diversity of tambjamines, including tambjamines A (**21**), C (**22**) and D (**23**) (Figure 3), increased from the bryozoan *Virididentula dentata* to its nudibranch predators *Tambja stegosauriformis* and *T. brasiliensis*. The highest tambjamin diversity and concentration were also observed in the carnivorous nudibranch *Roboastra ernsti*, which preys on *T. stegosauriformis* and *T. brasiliensis*, probably due to biomagnification [54].

Commonly known as shipworms, Teredinidae are marine bivalves highly adapted to a wood-feeding and wood-boring lifestyle. Their success in exploiting wood as a new food source is facilitated by the acquisition of cellulotytic gammaproteobacterial endosymbionts found in bacteriocytes within the gills of the invertebrates. The γ-proteobacterial symbiont was eventually identified as a new genus and species, *Teredinibacter turnerae* T7901, isolated from the gill tissue of a wood-boring shipworm, *Lyrodus pedicellatus* [55]. In addition to its ability to break down cellulose, *T. turnerae* can fix dinitrogen as well as grow under culture conditions. The ability to fix nitrogen is important as it provides a source of fixed nitrogen for the animal in a highly nitrogen-limiting environment. Moreover, the bacterial-derived cellulase enzyme is selectively transported from the gills to the shipworm’s bacteria-free gut, cecum, where the enzyme can digest cellulose to liberate energy-rich glucose for the animal [56]. This novel digestion strategy contrasts with other symbiotic relationships where the microbial symbionts are found in the gut and where the breakdown of cellulose results in the release of nutrient-poor products such as acetate.

**Figure 4:**
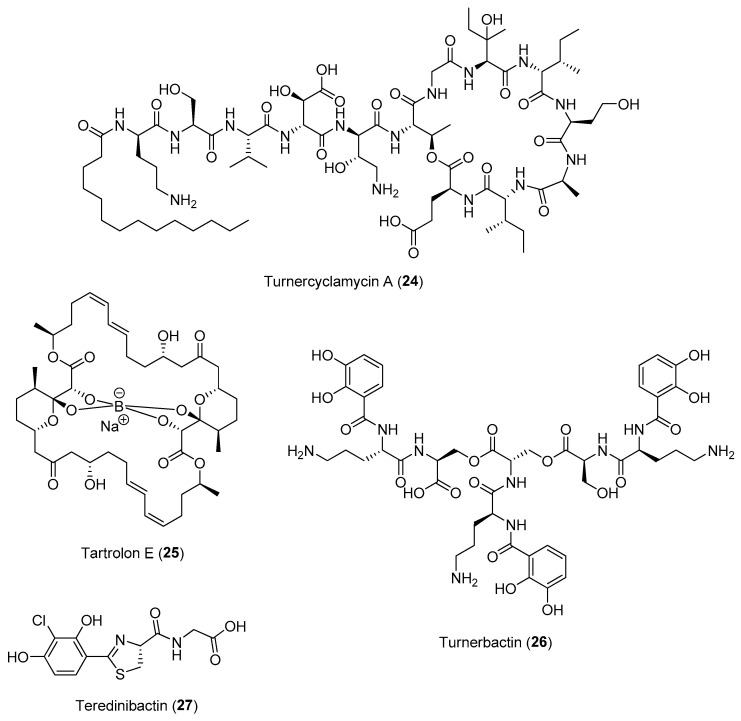
Specialized metabolites from *Teredinibacter turnerae* T7901.

Interestingly, genomic sequencing data revealed that a large proportion of the genome of *T. turnerae* encodes enzymes related to secondary metabolism (about 7%), comparable to that of the biomedically important *Actinobacteria* [57]. This could be due to the adaptation of the symbionts to be hosted in the animal’s gill by secreting potent antibiotics instead of the digestive organ. Coupled with the ability of large-scale fermentation of the bacteria, a series of bioactive compounds, including turnercyclamycins (e.g., **24**), boronated tartrolon E (**25**), turnerbactin (**26**) and teredinibactins (e.g., **27**), were subsequently reported from *T. turnerae* (Figure 4). Both turnerbactin, a triscatecholate siderophore, and the phenolate-thiazoline containing teredinibactins have high metal-chelating properties for the regulation and uptake of metals such as iron, copper and molybdenum for both the host and symbiont as well as for the limiting of the growth of microbial pathogens via sequestration of iron [58,59]. The potent antibiotic polyketide tartrolon E (**25**) as well as the cyclic peptides known as turnercyclamycins are produced possibly to ward off microbial competitors in the gills and in the cecum to prevent other microbes from using glucose, the product of cellulase degradation [60,61]. Moreover, both turnercyclamycins and tartrolon E were found active against colistin-resistant *Acinetobacter* and apicomplexan parasites, respectively. Tartrolon E has been hypothesized to function as a chemical defense that protects the host animal from gregarines, a diverse group of ancestral apicomplexan parasites known to be pathogens in mollusks. Subsequent bioassay-guided fractionation has identified tartrolon E as exhibiting a nanomolar-to-picomolar range activity against a panel of apicomplexan parasites, including *Cryptosporidium*, *Plasmodium*, *Babesia*, *Theileria* and *Sarcocystis* [62]. For instance, tartrolon E has an EC_50_ of 3 nM against *T. gondi,* and it kills the parasites within 2 h of treatment. These series of initial studies on shipworm–bacteria interactions reveal bacterial symbionts to be a treasure trove of bioactive natural products. Recent metagenomic studies on other species of shipworms, such as *Neoteredo reynei*, revealed that symbiotic microbiomes of shipworms are a rich untapped source of not only biomedically important secondary metabolites, but also biotechnologically relevant enzymes [63].

**Figure 5:**
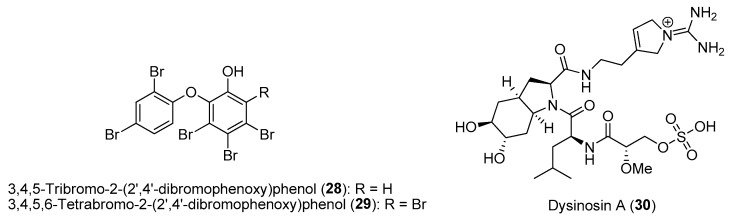
Specialized metabolites involved in chemical defenses mediated by marine invertebrate-associated microbial symbionts.

Mutualistic relationships between hosts and microbes involving exchanges of nutrients are common in nature. For instance, symbiotic cyanobacteria are known to provide fixed carbon in exchange for protection and nitrogen from the host animals. These microbial symbionts can also produce bioactive natural products as a chemical defense for the host animals [64]. One such example is the common marine sponge *Lamellodysidea herbacea*, harboring the filamentous cyanobacterial symbiont *Hormoscilla spongeliae* (formerly *Oscillatoria spongeliae*), which is found in high abundance in the sponge mesohyl matrix. The sponge, *L. herbacea*, is chemically talented in its bioactive natural products, with polybrominated diphenyl ethers (PBDEs) being the best-known class of molecules. These PBDEs, such as 3,4,5-tribromo-2-(2′,4′-dibromophenoxy)phenol (**28**) and 3,4,5,6-tetrabromo-2-(2′,4′-dibromophenoxy)phenol (**29**), have been shown to be biosynthesized by the cyanobacterial symbionts, and their production can account for more than 10% of the sponge’s dry weight (Figure 5) [65]. Due to the symbiont’s lack of essential pathways, including genes involved in histidine, thiamine and biotin biosynthesis, they are not amenable to laboratory culturing and are considered as obligate microbes. The host sponge depends on symbiont fixation, as field studies revealed that the inhibition of cyanobacteria photosynthesis by shading led to a higher mortality rate of *Lamellodysidea* sponges [66]. In addition to the diverse pharmacological activities, such as antimicrobial, cytotoxicity and enzyme inhibitory properties, several PBDEs have been shown to possess ecological functions [67]. Recent research by Faisal and co-workers demonstrated that **28** and **29** confer chemical defense on the sponge host against fish herbivory by the pufferfish *Canthigaster solandri* and on panels of environmentally relevant pathogenic and non-pathogenic bacterial strains [68]. Such ecological functions of PBDEs could explain the widespread occurrence of the sponge in Indonesia and the Indo-Pacific region.

Despite the cyanobacterial symbiont’s resistance to culturable conditions, the use of cell enrichment techniques coupled with advances in hybrid gene sequencing and assembly methods can facilitate detailed genomic analysis. For instance, based on the comparative genomics of cyanobacterial symbionts, two phylogenetically distinct *Hormoscilla* symbionts obtained from two chemotypes of *L. herbacea* have been shown to habour the biosynthetic gene for either PBDEs or dysinosin nonribosomal peptides, e.g., dysinosin A (**30**) (Figure 5) [69]. This study shed light on the concise strategy for the sponge host to obtain chemical diversity by associating with one dominant cyanobacterial strain as oppose to having a diverse community of natural product-producing microbes. Moreover, the generation of high-quality genomic information, based on advances in hybrid sequencing, can provide insights into microbial symbiotic lifestyle and uncover their potential for bioactive specialized metabolite production.

#### 2.1.3. Chemical-Mediated Defenses in Marine Holobionts

**Figure 6:**
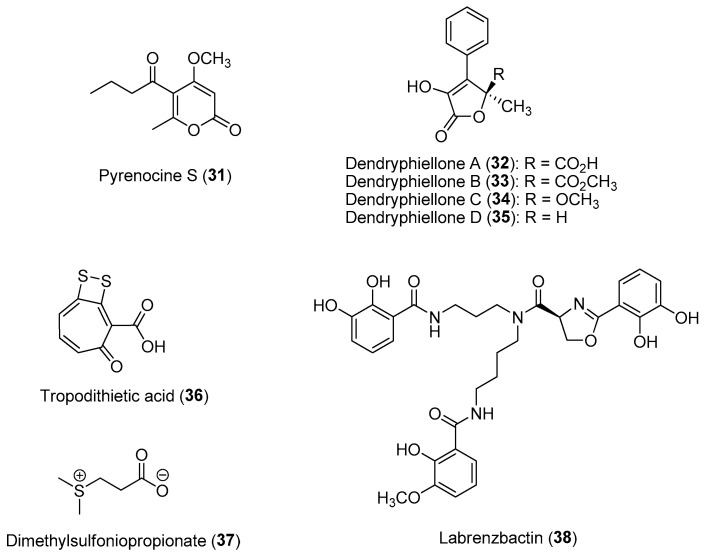
Compounds involved in chemical defenses in marine holobionts.

As illustrated in the above section, marine microbes associated with eukaryotic host organisms, including macroalgae and invertebrates, are usually the biogenetic sources of many bioactive natural products with important ecological functions. These host-specific microbial assemblages, including bacteria, fungi, viruses, protozoa and cyanobacteria, are diverse and can be found on the surface or residing within the host organisms. The study of host–microbiota interactions, termed the holobiont, can provide information on their crucial roles related to the structure and function of marine ecosystems. This is especially relevant in marine environments since microbes constitute the majority of the biomass and can influence biogeochemical processes. For example, sponge-associated microbial symbionts are able to convert dissolved organic carbon released by reef organisms into particulate organic carbon as food for heterotrophic organisms [70]. These microbial consortia can respond and adapt to the host developmental stage and its conditions due to changing environmental conditions and structures. For instance, chemical ecological studies on epiphytic microbial communities found on macroalgae are known to play fundamental roles in the regulation and maintenance of host fitness related to ecophysiology and metabolism. In addition, core epiphytic bacterial communities can provide macroalgal surfaces with protection from biofouling, pathogenic attacks and grazers through the production of bioactive molecules [71]. A study on 71 heterotrophic bacteria isolated from the green alga *Ulva rigida* revealed that 36% of the bacterial isolates showed antimicrobial properties, whereas such activity was not present in free-living bacteria obtained from the surrounding water [72]. Microbial endophytes found living within the tissues of macroalgae can also confer protection to the macroalgal hosts from marine pathogens. For example, a study on the chemistry of cultivable fungal endophytes from the brown alga *Ectocarpus siliculosus* revealed the production of new pyrenocines, e.g., pyrenocine S (**31**) (Figure 6), by the fungal strain *Phaeosphaeria* sp. AN596H to reduce infection by protistan pathogens [73]. In addition, a series of novel antimicrobial α-hydroxy γ-butanolides, dendryphiellones A (**32**) to D (**35**) (Figure 6), were isolated from the obligate marine fungus *Paradendryphiella salina*, obtained from several healthy brown macrophyte host species, such as *Saccharina latissima*, *Laminaria digitata*, *Pelvetia canaliculata* and *Ascophyllum nodosum* [74]. These defensive dendryphiellones were found to interfere with the bacterial quorum sensing system, based on bioassays with the pathogenic bacterial strain *Pseudomonas aeruginosa*.

Coral-associated microbes, particularly bacteria found living in the surface mucus layer of corals, are prolific sources of anti-fouling, cytotoxic and antimicrobial molecules, possibly acting as a first line of defense to protect the coral host against pathogens and to prevent overgrowth by invasive microbial biofilms [75]. Not only do coral mucuses confer chemical protection to the invertebrate host, but a recent study also suggested that dolphins utilize them as medicine [76]. It was revealed that the antifouling compounds identified from coral-associated microbes have a higher potency than those obtained from free-living microbes. Due to the apparent lack of an adaptive immune system, it was hypothesized that corals select microbes from the environment to allow the host animal to adapt to their surroundings [77]. For example, an investigation into the 156 different bacterial residents found in the mucus of the stony coral *Oculina patagonica* revealed that nine bacterial strains inhibited the growth of a coral pathogen, *Vibrio shiloi* [78]. Similarly, a bacterial strain, *Pseudovibrio* sp. P12, isolated from the coral *Pocillopora damicornis* is able to form tropodithietic acid (**36**), likely derived from dimethylsulfoniopropionate (**37**) catabolism, having strong antibacterial activity against the coral pathogens *Vibrio coralliilyticus* and *V. owensii* (Figure 6) [79]. Dimethylsulfoniopropionate is an important compound that plays an essential role in the marine sulfur cycling process. It is also involved in several cellular and ecological functions, including antioxidant properties, cryoprotectant in marine algae, antiviral defense mechanisms and sulfide detoxification [80]. Labrenzbactin (**38**) is another compound recently reported from a *Montipora*-sp.-associated bacterial strain, *Labrenzia* sp., which has antagonistic interactions with pathogens (Figure 6) [81]. Based on the molecule’s ability to inhibit the growth of the Gram-negative plant pathogen *Ralstonia solanacearum* and the Gram-positive *Micrococcus luteus*, it is speculated that it could provide broad-spectrum antimicrobial protection to the coral host. As such, invertebrate/macroalgal holobionts represent unique models for the discovery of novel bioactive compounds as well as for strategies to combat the rise and spread of antimicrobial resistance [82,83]. A common feature of antimicrobial compounds isolated from marine bacteria obtained from the surfaces of macroalgae and corals is in their ability to inhibit competing Gram-negative bacteria through interference with bacterial quorum-sensing systems [84,85]. In fact, up to a quarter of the bacteria associated with seven stony coral species and one soft coral species from the Gulf of Eilat, Israel exhibited quorum-sensing inhibitory properties [86].

The sponge holobionts consist of diverse and abundant microbial symbionts, including Proteobacteria, Acidobacteria, Chloroflexi, Cyanobacteria and the sponge-specific Poribacteria [70]. These symbiotic microbes are known to provide their sponge hosts with defensive molecules to avoid predation, fouling and infection. In turn, the microbial symbionts receive primary metabolic nutrition and a hospitable environment within the host animals. Microbial densities are found to vary significantly across sponge species and can be classified into two distinct symbiotic community states, namely high microbial abundance (HMA) and low microbial abundance (LMA) [87]. LMA symbiotic state is the ancestral state among sponges, while HMA symbioses, consisting of higher specialized microbes, have evolved numerous times through the recruitment of similar assemblages. Furthermore, recent studies have found that HMA holobionts have higher endemism and metabolic dependence and encode for secondary-metabolite biosynthesis in chemical defenses [88,89].

**Figure 7:**
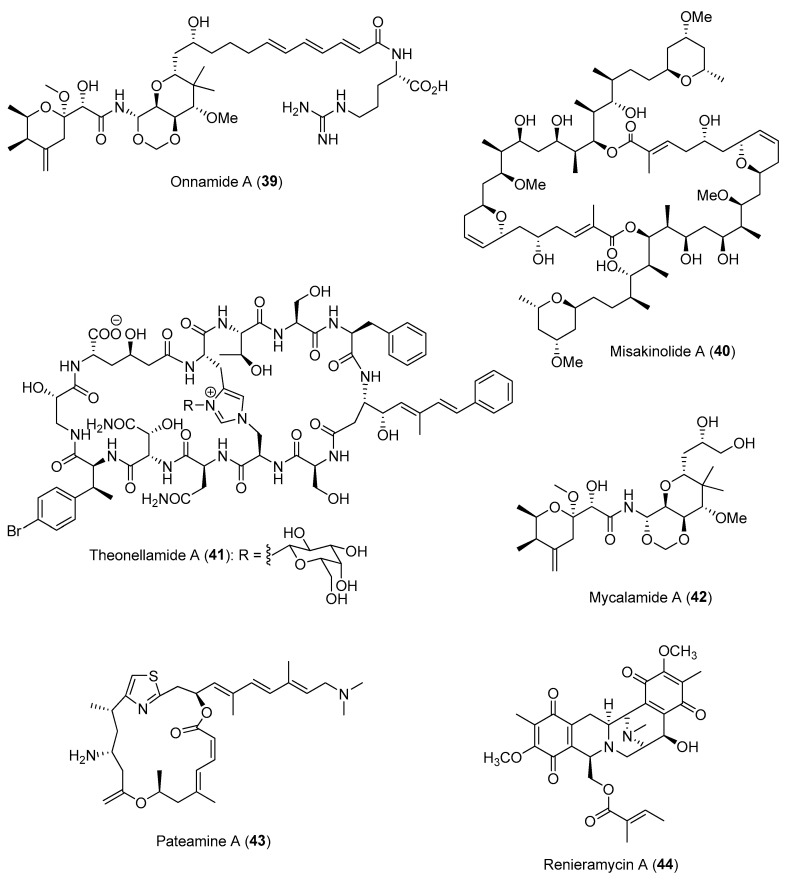
Specialized metabolites involved in chemical defenses in sponge holobionts.

In some cases, the interdependent relationship between the sponge host and the microbes could lead to the maintenance of specific microbial ‘super producers’ with high numbers of biosynthetic gene clusters. For instance, the uncultivated filamentous bacterium “*Candidatus* Entotheonella” was discovered to produce almost all the defensive molecules—including polyketides such as onnamide A (**39**) and misakinolide A (**40**) and peptides such as theonellamide A (**41**)—previously isolated from sponges of the genus *Theonella* (Figure 7) [90,91]. In contrast, multiple genera of endosymbiotic microbes, namely “Entomycale ignis”, “Patea custodiens” and “Caria hoplite”, which were found to be associated with the New Zealand sponge, *Mycale hentscheli*, were found to cooperatively contribute to an array of defensive molecules, including mycalamide A (**42**), pateamine A (**43**) and polytheonamide-type gananamides of the holobiont (Figure 7) [92]. The production of these defensive microbial chemicals can be found in specialized sponge cell types, such as the renieramycin-producing renieramycin A (**44**) (Figure 7) and symbiont Ca. Endohaliclona renieramycinifaciens, found localized in the chemobacteriocytes of *Haliclona* sponges [93]. Interestingly, the genome of Ca. E. renieramycinifaciens has undergone extreme reduction, losing almost all necessary genes for free living and receiving essential nutrients from the sponge host. Moreover, Ca. E. renieramycinifaciens avoid other competing bacteria by residing in specialized chemobacteriocytes. The notion that marine natural products originated from microbial symbionts and not from the animal hosts has significant implications for drug discovery, as it allows for a bacterial-based production system.

In summary, ecological studies on host-microbial interactions and their metabolomes could advance our understanding of marine symbioses and allow greater utility of these symbionts in drug discovery and biotechnological applications. Studies could provide insights and approaches for enhancing the yield and expression of natural products from silent/cryptic biosynthetic gene clusters via elucidating essential signals for gene activation. This is potentially useful in addressing the supply issue of bioactive compounds as lead molecules, especially in the early stages of the drug discovery pipeline. In addition, previously unculturable microbes could be made amenable to cultivation with knowledge of their natural environment. To this end, several innovative technologies have been developed for cultivating the ‘uncultivated’, including single cells based on microfluidic technologies and isolation chips (iChip). Moreover, advances in omics approaches, including genomics, proteomics, transcriptomics and metabolomics, will continue to shed light on the intricacies of host-microbial interactions and their applications [94].

### 2.2. Activated/Induced Chemical Defenses

**Figure 8:**
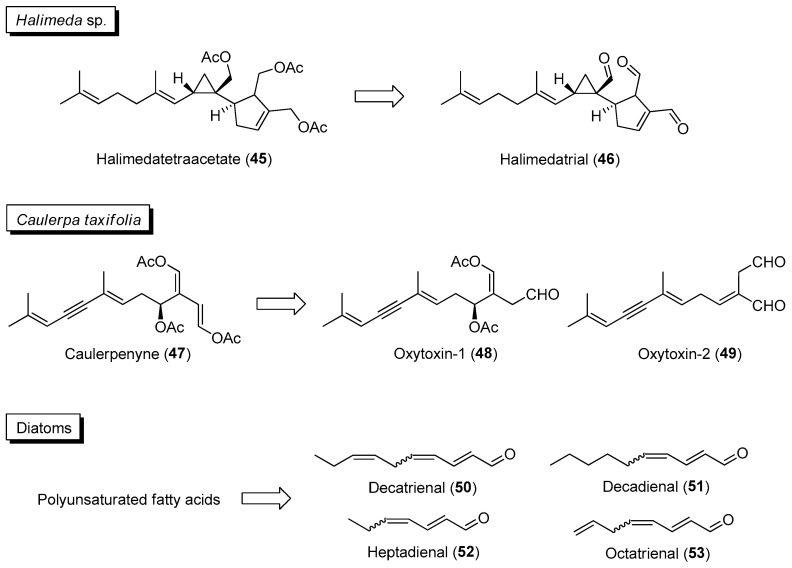
Specialized metabolites involved in activated chemical defenses.

Certain marine benthic and planktonic organisms, such as microalgae, macroalgae and invertebrates, employ activated defenses by chemically converting less-active molecules, stored within tissues, into bioactive analogues when they are being attacked or wounded. The production of more active compounds can also be induced by chemical cues associated with mechanical damage, herbivore-specific cues and feeding-specific cues. Knowledge of activated/induced defense mechanisms used by marine organisms can be exploited for the discovery of new therapeutic agents. For example, instead of the conventional extraction of fresh or freeze-dried marine samples that may result in the detection of reduced chemical diversity, samples can be “wounded” by mechanical methods to induce the formation of bioactive compounds. Moreover, the frequency of activated chemical changes has been observed within 30 s upon physical wounding in the seawater of certain marine samples such as temperate and tropical seaweeds [95]. An interesting example is the wound-activated formation of the potent feeding deterrent halimedatrial (**46**) from the diterpenoid halimedatetraacetate (**45**) in the green alga *Halimeda* sp. (Figure 8) [96]. Similar rapid chemical transformation has also been observed in the invasive green alga *Caulerpa taxifolia*, where the acetate groups of caulerpenyne (**47**) were enzymatically cleaved within minutes upon mechanical damage into reactive oxytoxin products **48** and **49** (Figure 8) [97]. Planktonic microalgae such as diatoms can undergo quick production of *α*,*β*,*γ*,*δ*-unsaturated aldehydes **50** to **53**, via the enzymatic cleavage of polyunsaturated fatty acid precursors, with anti-predator activities that negatively impact the reproductive success of their copepod predators (Figure 8). Moreover, the production of other bioactive products, such as oxylipins, can accompany the activated formation of polyunsaturated aldehydes in diatoms [98]. The activation/induction of these diatom-derived oxylipins, such as anticancer 2*E*,4*E*-decadienal and 2*E*,4*E*,7*Z*-decatrienal, can be explored for potential biomedical applications [99].

**Figure 9:**
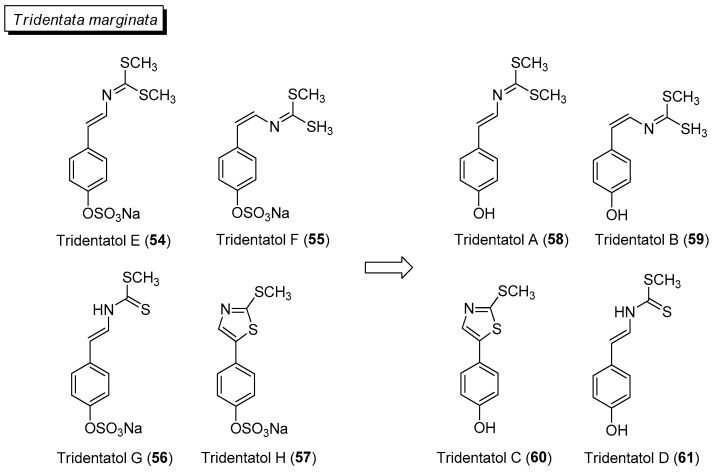
Specialized metabolites involved in activated chemical defense in hydroid.

Several marine invertebrate species have been reported to adopt activated defenses upon physical damage by predators. From the marine hydroid *Tridentata marginata*, a series of dithiocarbamates, tridentatols A (**58**)–D (**61**) are activated from tridentatols E (**54**)–H (**57**) upon tissue damage to provide chemical protection from fish predators (Figure 9) [100]. Interestingly, the antifungal tridentatol C is also biosynthesized under abiotic stress as a chemical defense in the crucifer watercrass *Nasturtium officinale* [101]. Tridentatol A (**58**) was reported to be a more potent antioxidant against the lipid peroxidation of low-density lipoprotein compared to vitamin E [102].

**Figure 10:**
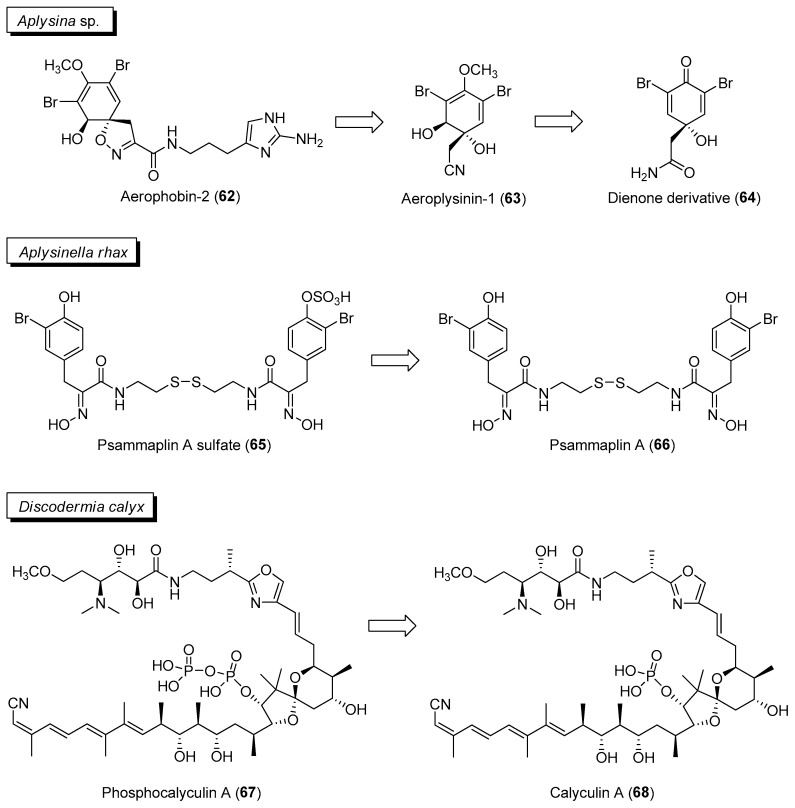
Specialized metabolites involved in activated chemical defenses in sponges.

An activated chemical defense mechanism has been reported to be prevalent in tropical sponges to confer protection to the animal from microbial pathogens [103]. For instance, in sponges belonging to the genus *Aplysina*, isooxazoline alkaloids such as aerophobin-2 (**62**) are converted to antimicrobial aeroplysinin-1 (**63**) and a dienone (**64**) derivative upon wounding (Figure 10) [104]. Subsequent biological evaluation of aeroplysinin-1 revealed its potential use for the treatment of different pathologies, including inflammation, cancer and bacterial infection [105]. Similarly, a related sponge, *Aplysinella rhax*, is able to convert the ecologically less active psammaplin A sulfate (**65**) to psammaplin A (**66**) when sponge tissue was stabbed or ground for one minute (Figure 10) [106]. This is supported by feeding experiments with an omnivorous sponge predatory pufferfish, *Canthigaster solandri*, where the converted molecule, psammaplin A, displayed antifeedant activity compared to the precursor psammaplin A sulfate. The bromotyrosine derivative, psammaplin A (**66**), is a well-studied molecule and is found to have a broad-spectrum biological activity ranging from antimicrobial to antiproliferative properties [107,108]. A paper by Thoms and Schupp suggested that there could be a predisposition of tyrosine-derivatives for activated defenses since the precursors of tridentatols, aeroplysinin-1 and psammaplin A, belong to this structural class [106].

Activated defense can also be mediated by microbial symbionts associated with the animal host. A study by Wakimoto and co-workers revealed the biosynthetic gene cluster of the cytotoxin calyculin A (**68**) (Figure 10) to be in an uncultivated bacterial symbiont, “*Candidatus* Entheonella” sp., found associated with the sponge, *Discodermia calyx* [109]. Calyculin A (**68**), a known potent inhibitor of serine-threonine phosphatases PP1 and PPA2, is used as a chemical tool to study cellular signaling pathways. Upon host injury by predators, calyculin A (**68**) is formed from the phosphorylated protoxin phosphocalyculin A (**67**) (Figure 10) via a microbial enzyme to confer protection to the host animal [110]. Moreover, storage of the less active phosphocalyculin A prevents self-toxicity in the marine sponge.

Induced defenses through the increased production of certain metabolites have been demonstrated in several marine organisms, including seaweeds, sponges and phytoplanktons [103,111,112,113]. Laboratory experiments on the red alga *Laurencia dendroidea* revealed an increased concentration of elatol (**114**) for up to 2 days in response to simulated herbivory [112]. An induced defense was also observed in the brown alga *Fucus vesiculosus*, where temporal variability in induced traits of the alga as well as changes in the expression of genes with putative defensive functions were observed [111,114]. In another red alga, *Pyropia haitanensis*, Chen and co-workers showed that an oxylipin, 1-octen-3-ol acts as a chemical messenger that primes the alga to upregulate the biosynthesis of several defensive compounds, including oxylipins, methyl jasmonic acid, indole-3-acetic acid and gibberellin A3, resulting in host defense induction [115].

### 2.3. Allelochemicals in Competition for Space and Resources

**Figure 11:**
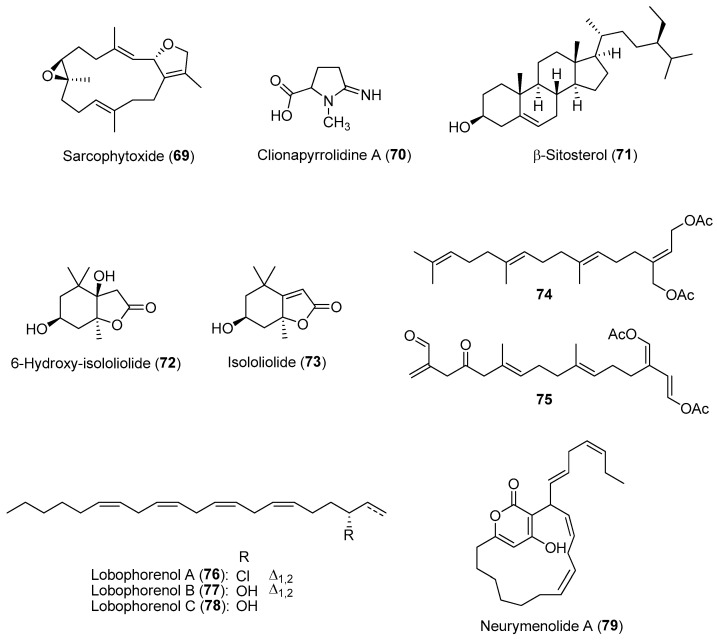
Allelochemicals in competition for space and resources.

In marine reef habitats, space is a limiting factor, particularly on hard substrates found in the photic zone where sufficient light penetrates the water column to allow photosynthesis. Due to the high density of sessile benthic organisms found within this zone, they evolved allelopathic interactions to compete for limited space and resources. In addition to allelochemicals, marine benthic invertebrates such as poriferans, cnidarians, tunicates and bryozoans use a variety of alternative mechanisms, including early sexual maturity, higher growth and fecundity rates, as well as the presence of sweeper polyps to defend their occupied area. Many allelochemicals used during competition are potential cytotoxic compounds as they cause tissue necrosis and cell damage to prevent/arrest the growth of nearby competitors [9]. For instance, soft corals are known to produce structurally diverse terpenoids with antifouling and cytotoxic properties [116]. The soft coral *Sarcophyton glaucum* was investigated for its natural product variation as well as its competitive interactions with the scleractinian coral *Acropora robusta*. In field experiments, high concentrations of the diterpene sarcophytoxide (**69**) (Figure 11) were found in female soft coral colonies that were placed in contact with *A. robusta* [117]. Sarcophytoxide, along with three other soft coral metabolites, were recently found to possess proteasome inhibitory and cytotoxic (against MCF-7 breast cancer) activities [118].

Sponges are common benthic invertebrates and are considered as one of the top spatial competitors on reef systems. Their success is, in part, due to their ability to utilize a wide range of nutritional sources as well as diverse chemical defenses against predators, microbial attacks and sessile benthic competitors. It was revealed that sponge bioactive allelochemicals are implicated in the increasing abundance of animals on reefs in Zanzibar [119]. Of the ten dominant sponges found on reefs in Zanzibar, 80% of the sponge-derived crude extracts showed antimicrobial activity, while seven sponge species, including *Callyspongia* sp. and *Scopalina hapalia*, exhibited cytotoxic properties based on the brine shrimp toxicity assay. Specific sponge-derived allelochemicals have also been identified to confer a competitive edge in interactions with other marine invertebrates. In a study involving the excavating sponge *Cliona tenuis*, clionapyrrolidine A (**70**) (Figure 11) was found to be toxic to scleractinian corals, killing coral tissue upon contact and lowering the photosynthetic potential of coral zooxanthellae [120]. The steroidal b-sitosterol (**71**) (Figure 11), isolated from another sponge species such as *Cinachyrella* cf. *cavernosa*, was shown to cause bleaching of the aggressive zoanthids *Zoanthus sansibaricus*, possibly by affecting the zoanthid’s symbiotic zooxanthellae [121].

Macroalgae are chemically rich marine plants known to produce inhibitory lipid-soluble allelochemicals used to compete with other benthic organisms, particularly cnidarians, for space in reef systems. These allelochemicals are usually produced by specialized cells and localized onto the macroalgal surfaces. As such, contact is usually required for these molecules to exert an allelopathic effect on potential competitors [122]. Such allelopathic interactions may suppress/limit coral reef recovery due to the proliferation of macroalgae in constant contact with remaining corals. The chemical structures of allelochemicals have been reported in several macroalgal species, using traditional extraction and bioassay-guided purification methods. Several terpenoids, including loliolide derivatives such as **72** and **73** and acetylated diterpenes such as **74** and **75**, isolated from two macroalgae, *Galaxaura filamentosa* and *Chlorodesmis fastigiata*, respectively, were identified as potent allelochemicals against the corals *Montipora digitata*, *Acropora millepora*, and *Pocillopora damicornis* (Figure 11) [123]. These hydrophobic allelochemicals could exert their allopathic effects by impacting coral photosystems, coral bleaching, and coral deaths via their transfer from macroalgal surfaces. In another in situ study on several brown algal species belonging to *Lobophora* genus, three macroalgal C21 polyunsaturated alcohols, lobophorenols A (**76**)–C (**78**) (Figure 11), were responsible for causing bleaching in some coral species, particularly *Acropora muricata*, upon direct contact [124]. In addition, an *α*-pyrone macrolide allelochemical, neurymenolide A (**79**) (Figure 11), was found on all surfaces of the red macroalga *Phacelocarpus neurymenioides*, especially on the basal portions of the algal blades, using desorption electrospray ionization mass spectrometry [125]. This molecule was found to induce bleaching via contact of the macroalga on the natural population of *Porites rus*. Previously isolated from another red macroalga—*Neurymenia fraxinifolia* (from Fiji), neurymenolide A is found to possess antibacterial and cytotoxic property by inhibiting formation of the mitotic spindle in U-2 OS osteosarcoma human cells [126].

A range of allelochemicals are also employed by bloom-forming marine microbes, such as cyanobacteria and dinoflagellates, to compete with sympatric macrophytes, algae and other microbes. These allelochemicals can also have defensive roles against potential predators and grazers. Allelochemicals can impact target organisms negatively by inhibiting photosynthetic processes, causing cell membrane lysis, enzymatic inhibition, and the prevention of RNA synthesis and DNA replication, inducing temporary cyst formation and blocking cell motility and division [127]. The reasons that these defensive allelochemicals can target mammals, including humans, could be due to their ecological interactions with similar systems found in competing micro/macroorganisms. It is therefore not surprising that numerous compounds isolated from marine cyanobacteria are found to possess antiproliferative properties with specific molecular targets, such as enzymes, microtubules and tubulin, as well as sodium channels [128]. However, microalgal allelochemicals are usually produced in minute quantities, which can be challenging in terms of their detection and eventual chemical determination. These allelochemicals are not only sources of potential pharmaceuticals, but also as algaecides, herbicides and insecticides [129]. Since the extensive coverage of microalgal allelochemicals is beyond the scope of this review, readers can refer to several excellent reviews on this topic for more information [3,129,130,131,132,133,134,135]

### 2.4. Allelochemicals in Phycosphere of Phytoplanktons

The interaction between phytoplankton and bacteria is an important inter-organism association in the aquatic environment. Such association can significantly influence global carbon and nutrient cycling and regulate the aquatic food web. The mucus interface between algal cells and bacteria, known as phycosphere, is a region rich in metabolites and infochemicals that mediates a host of physiological phenomena, such as mutualism, commensalism, antagonism, parasitism and competition [136]. This microenvironment consists of a high concentration of bacterial cells (measuring between 0.2 and 2 mm) surrounding the algal host (about 2 to 200 mm). Within the phycosphere region, communication between microalgae and bacteria depends on a variety of lipid-based compounds that can cross microbial membranes and trigger a range of metabolic responses.

**Figure 12:**
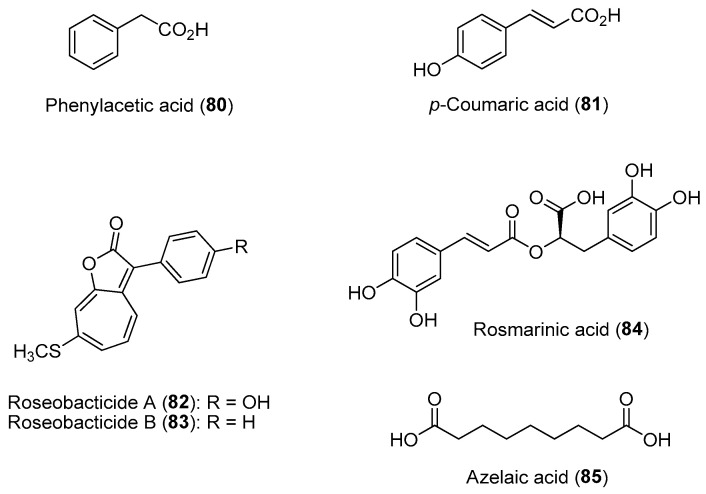
Allelochemicals in phycosphere of phytoplanktons.

Extensive studies have been carried out on the chemical communication between the bloom-forming eukaryotic microalga *Emiliania huxleyi* and the α-proteobacterium *Phaeobacter gallaeciensis* association. Studies revealed that *P*. *gallaeciensis* produces molecules that include broad-spectrum antibiotics such as tropodithietic acid (**36**) and growth-promoting compounds such as auxin phenylacetic acid (**80**) to prevent the growth of pathogenic bacteria and promote algal growth, respectively (Figure 12) [137]. In turn, the algal host provides the bacterial symbionts with essential nutrients and a surface for colonization and biofilm formation. However, the mutualistic association flips to a parasitic lifestyle for the bacterial symbiont upon sensing the senescence of the algal host. Specifically, the ageing algal cells release a lignin breakdown product, *p*-coumaric acid (**81**), which is utilized by the parasitic *P*. *gallaeciensis* to produce algicides such as roseobacticides A (**82**) and B (**83**). The eventual death of the algal cells, caused by the bacterial-derived algicides, releases nutrients to be used by the bacteria and are dispersed to healthier and younger algal hosts in the bloom [138].

Using an integrated multiomics approach, a recent study by Shibl and co-workers showed that the ubiquitous unicellular eukaryotic diatom *Asterionellopsis glacialis* can modulate its association with bacteria using two unusual diatom-derived natural products, rosmarinic acid (**84**) and azelaic acid (**85**) (Figure 12) [139]. Using these two molecules, the diatom is able to select and promote the attachment of beneficial bacterial taxa while suppressing the colonization of opportunistic bacterial types. Rosmarinic acid is also produced by the plant *Arabidopsis thaliana* and is a homoserine lactone mimic able to bind to the pathogenic bacterium *Pseudomonas aeruginosa* quorum-sensing regulator RhlR [140]. It is possible that the diatom-derived rosamarinic acid controls bacterial motility and attachment via interference with the bacterial quorum-sensing system. Moreover, exposure of the host diatom to its natural microbial community resulted in significant transcriptional and metabolic changes, leading to the release of these unique metabolites. These examples of microalgal–bacterial interactions highlight the complex and intricate nature of chemical communications, and deciphering these functional compounds at the molecular level requires innovative technologies. Current knowledge has revealed that quorum sensing and quorum quenching, based on small organic molecules, regulate a balancing act between a symbiotic and a parasitic way of life between phytoplankton hosts and associated bacteria [141,142]. Functional approaches to the identification of molecules involved in these microbial interactions can provide insights to analogous systems and facilitate the discovery of potential molecular tools for the treatment and prevention of human diseases such as microbial infections [143].

### 2.5. Phylogeny-Based and Concerted Discovery Strategies

The integration of molecular and analytical chemical techniques and the targeting of species belonging to divergent evolutionary lineages could increase the likelihood of finding highly divergent novel natural products. Such an approach, known as the “Concerted Discovery Strategy”, was adapted for the discovery of new peptide toxins, teretoxins, from the family Terebridae, a group of predatory marine gastropods classified within the superfamily Conoidea, comprising about 4000 species. The importance of conoidean toxins has been realized with the development of an analgesic peptidic conotoxin drug, ziconotide, from the *Conus* sp. (family Conidae) and is used for pain treatment in HIV and cancer patients. Teretoxins are structurally similar to conotoxins, and they represent new therapeutics that target ion channels and receptors [144].

**Figure 13:**
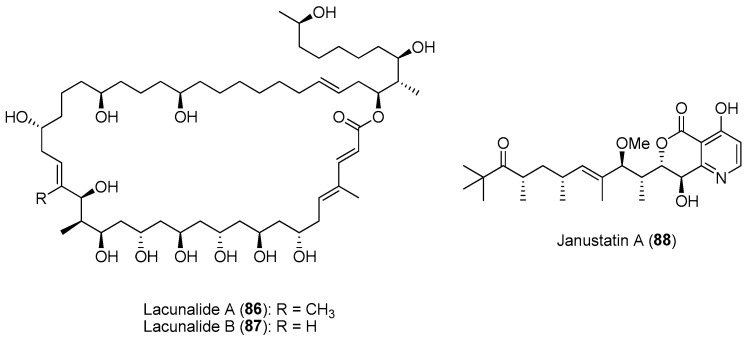
Specialized metabolites produced by *Gynuella sunshinyii*.

Studies show that exploring phylogenetically distinct or neglected clades could provide a greater potential for the discovery of novel molecules compared to well-known producer clades. For instance, there is currently significant phylogenetic biasness in the search for bioactive compounds from octocorals. Based on the assignment of 228 octocoral species that were investigated for their bioactive compounds, it was revealed that 90.8% of prospected soft coral species belonged to the *Holoxonia-Alcyoniina* clade, while only 7.4% and 1.8% were allocated to the *Calcaxonia-Pennatulacea* clade (e.g., sea pens) and the *Anthomastus-Corallium* clade (e.g., red corals), respectively [145]. As such, future bioprospecting could focus on these underexplored octocoral clades, including the *Calcaxonia-Pennatulacea* and *Anthomastus-Corallium* clades. Recent study on genome mining analysis revealed high biosynthetic potential for *Gynuella sunshinyii*, a chemically poorly studied plant-root associated Gram-negative marine bacterium from the underexplored order Oceanospirillales [146]. Subsequent chemical analysis resulted in the isolation of novel metabolites, including lacunalides A (**86**) and B (**87**) and janustatins (e.g., **88**) (Figure 13) having antibiotic and potent cytotoxic activities [146,147].

A retrospective analysis based on 25 years of Australian marine bioresources collection and research conducted by the Australian Institute of Marine Science provided a new chemical ecology rationale for drug bioprospecting [148]. The analysis revealed that high-level phylogeny, including deuterosome phylogeny and their ancestors (e.g., Porifera and Cnidaria), along with available metabolic machinery, accounted for the observed high bioactivities. Moreover, habitat diversity and ecological factors are important drivers in the activation of biosynthetic gene clusters present in these marine organisms. As no correlation between biogeographic bioactivity and biodiversity hotspots were found, the analysis showed value in the exploration of similar taxa occurring in diverse ecological habitats.

### 2.6. Spatial and Temporal Chemical Variation of Natural Products

**Figure 14:**
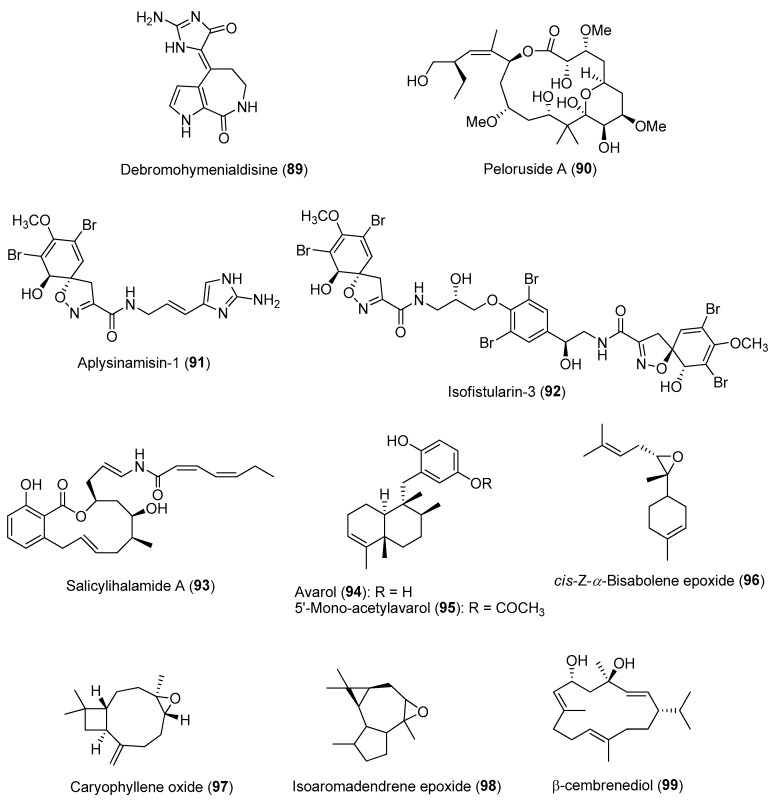
Spatial and temporal chemical variation of specialized metabolites.

Biotic and abiotic factors can affect the production and variation of bioactive natural product composition in marine organisms in nature. This could be due to selective pressures affecting the organism’s ability to produce secondary metabolites both qualitatively and quantitatively. Knowledge of the variation in natural products’ production could therefore be exploited for the discovery of novel bioactive compounds. Studies on the spatial variability of secondary metabolites within a marine invertebrate species, such as sponges, are not common and can be controversial [149]. Some studies showed no or low variability while others revealed significant variability in sponge natural product concentrations. For instance, extensive investigation into the chemistry of the Mediterranean sponge *Spongia lamella*, and specimens collected over an area of 1200 km revealed that chemical diversity differed greatly between sponge populations [150]. A similar trend was observed for another common sponge species, *Stylissa massa*, collected over different locations from local to ocean basins [151]. The concentration of alkaloids such as debromohymenialdisine (**89**) (Figure 14) showed significant variation among individuals from locations that spanned the Pacific basin. Concentrations of sponge-derived secondary metabolites, such as pateamine A (**43**) and peloruside A (**90**) from *Mycale hentscheli*, showed variations of depths (Figure 14) [152]. Other habitat variability, such as higher exposure to UV radiation at shallow/intertidal areas, can affects changes in the abundance of photosymbiotic species in sponges known to produce UV-protecting secondary metabolites such as mycosporine-like amino acids [153].

In addition to spatial patterns, temporal variations of compound concentrations have been reported in several sponge species. For instance, temporal variation in the production of brominated alkaloids, including aerophobin-2 (**62**), aplysinamisin-1 (**91**) and isofistularin-3 (**92**) (Figure 14), was observed in the marine sponge *Aplysina aerophoba* over a two-year period [154]. It was revealed that the high concentrations of the bromo-containing molecules found in the ectosome (outer) layer of the sponge corresponded to a relatively higher water temperature during the summer months. Similar observations were made on the concentration of the cytotoxic salicylihalamide A (**93**) (Figure 14) from the sponge *Haliclona* sp., found to be highest in summer [155]. In another study, variation in the concentrations of two sesquiterpene hydroquinones, avarol (**94**) and 5′-mono-acetylavarol (**95**) (Figure 14), were detected in the sponge *Dysidea avara* when they were placed in contact with marine invertebrates or algae [156]. In particular, avarol (**94**) showed significant intra-individual variation, wherein its high concentration was detected in the peripheral layer of the sponges when placed in close proximity to other sponge species. Furthermore, relatively high concentrations of both sesquiterpenes were detected in the spring and early summer, which correlated to the brooding season of *D. avara*.

A study on the antifouling activity of nine marine macroalgal extracts based on bimonthly samples from the Bay of Concarneau in France showed that almost 50% of the extracts displayed antifouling activity against at least one of the fouling organisms [157]. Of these, about one third of the extracts were seasonally active, with peak activity reported during the summer period. The antifouling activity of the extracts was tested against bacteria, fungi, diatoms, macroalgal spores and barnacle cypris larvae. The seasonal variation in antifouling activity of the selected macroalgae could be adaptive by coinciding with maximal pressure from the fouling communities within the bay. Similarly, extracts from the soft coral *Sarcophyton trocheliophorum* showed higher antifouling activity against fouling bacteria and barnacle larvae when collected during the summer season. Subsequent GC-MS analysis revealed higher concentrations of compounds such as *cis*-*Z*-a-bisabolene epoxide (**96**) and caryophyllene oxide (**97**) in summer soft coral samples, while isoaromadendrene epoxide (**98**) and *β*-cembrenediol (**99**) showed a higher peak area percentage in winter samples (Figure 14) [158].

**Figure 15:**
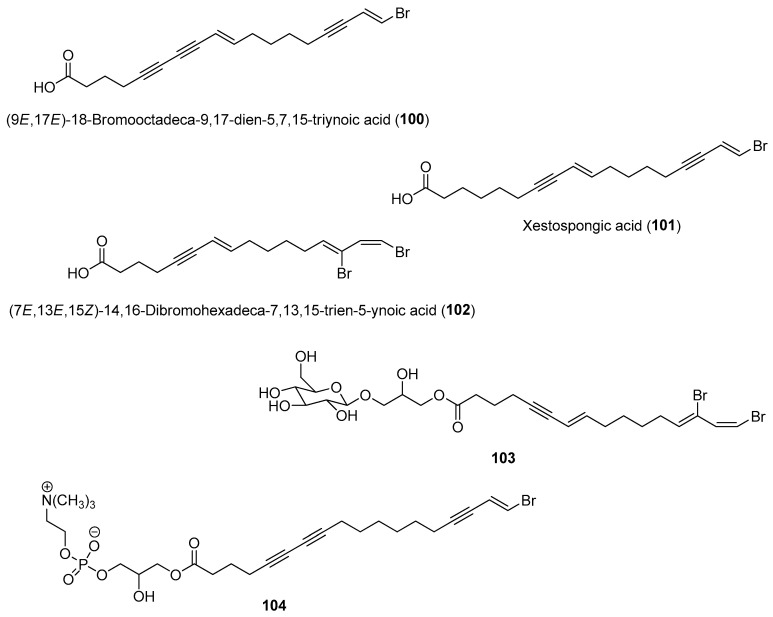
Chemical variation of specialized metabolites in *Xestospongia* sp.

Overall, the natural product chemistry of certain marine organisms exhibits significant temporal and spatial variations due to responses to biotic and abiotic factors. These variations in chemistry should be considered when evaluating certain benthic invertebrates for economic purposes, such as in aquaculture for drug production and development and in the discovery of new molecules [159]. Instead of a single sampling regime, a sampling program consisting of different months/seasons can be considered to capture the diverse metabolic profile and activity of a particular marine species. Such chemical variations could be detected using metabolomic techniques recently demonstrated for detecting changes in metabolic production in the Giant barrel sponge *Xestospongia* sp., collected from four collections in Martinique, Curaçao, Taiwan and Tanzania as well as two Mediterranean *Haliclona* sp. [160,161]. Based on metabolomics tools, including NMR and LC-MS, variations in sterols and fatty acids contents such as polyoxygenated and brominated derivatives were revealed in *Xestospongia* sp. samples collected from different locations. Moreover, brominated fatty acids, namely (9*E*,17*E*)-18-bromooctadeca-9,17-dien-5,7,15-triynoic acid (**100**), xestospongic acid (**101**), (7*E*,13*E*,15*Z*)-14,16-dibromohexadeca-7,13,15-trien-5-ynoic acid (**102**), and two previously unreported compounds (**103** and **104)**, were found to correlate with antibacterial activity when tested against *Escherichia coli* and *Staphylococcus aureus* (Figure 15) [161]. The majority of studies on the spatial and temporal changes of compound concentrations were confined to their quantitative analysis and lacked information related to the causing factors. Charting changes in microbiome diversity and relating them to chemical variations could provide insights into the possible microbial producers of bioactive secondary metabolites. More experimental studies are therefore necessary to vigorously prove the exact causes leading to metabolic variations in marine organisms.

## 3. Application of Innovative Techniques for Marine Chemical Ecology Research

Advancements in analytical technologies coupled with recent innovative metabolomic applications allowed for the revealing of low quantities of metabolites involved in organismal interactions, including symbiosis, defense and competition. The main advantages of these metabolomic-guided techniques over the classical bioassay-guided approach include the omission of a lengthy bioassay analysis after the subsequent separation step and the simultaneous detection of complex mixtures of active molecules. However, unlike the later approaches, which lead to the identification of actual bioactive molecules, metabolomic techniques can only predict biological activity. As such, metabolomic approaches should be coupled with bioassays to confirm the activity of candidate compounds. Moreover, metabolomic methods might not be able to detect molecules with natural concentrations below the detection limits of the analytical instruments. In spite of the inherent limitations of metabolomic methods, important information related to the ecological functions of molecules can be obtained [162].

**Figure 16:**
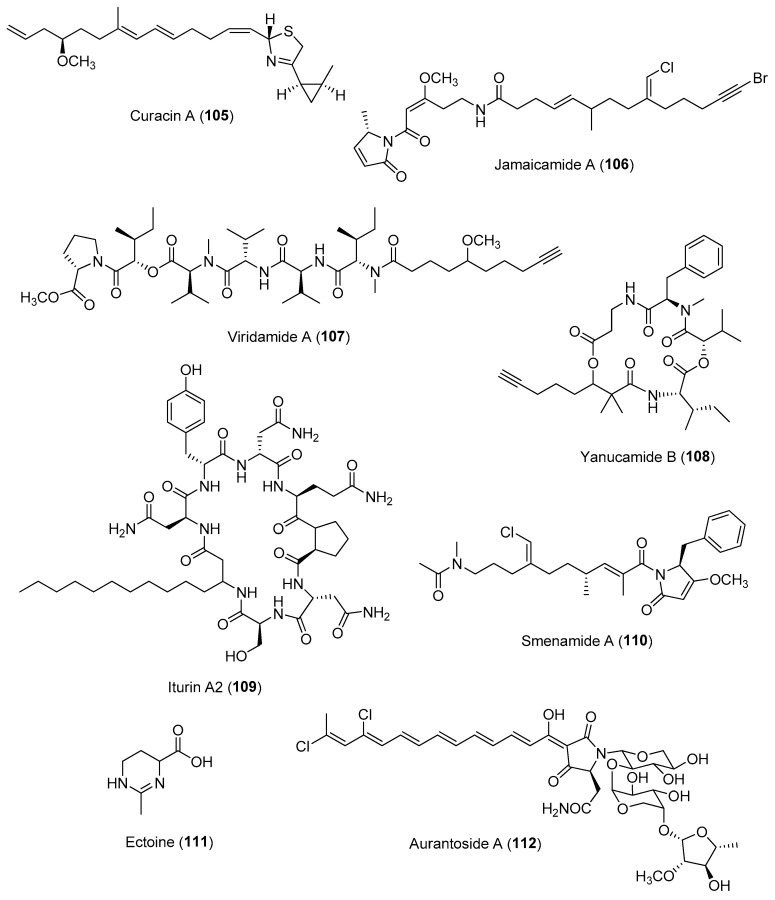
Specialized metabolites **105** to **112** detected by innovative analytical techniques.

Chemically mediated interactions between microbes or microbes and macroorganisms usually occur at the surface, as illustrated in the above sections. One of the recent analytical advances that allows for the analysis of surface chemistry is the development of analytical imaging MS (IMS), including desorption electrospray ionization (DESI) MS, matrix-assisted laser desorption ionization (MALDI) MS, matrix-free laser desorption ionization (LDI) MS, secondary ion MS (SIMS), droplet-liquid microjunction-surface sampling probe and laser-ablation electrospray ionization (LAESI) MS [163]. The main advantage of analytical imaging MS techniques is that they allow for spatial mapping on the distribution of allelochemicals such as defensive molecules on surfaces of biological samples, as demonstrated by pioneering studies on seaweeds and corals [28,164]. In particular, advances in MALDI-IMS beam technologies led to significant improvements in resolution to dimensions smaller than an average human cell [165]. MALDI imaging has been successful in identifying and linking several compounds, e.g., curacin A (**105**), jamaicamide A (**106**), viridamide A (**107**) and yanucamide B (**108**) (Figure 16), in a consortium of marine cyanobacteria, including *Lyngbya majuscula* 3L and JHB, *Oscillatoria nigro-viridis*, *Lyngbya bouillonii*, and a *Phormidium* species [166], a spatial distribution of specialized molecules in the marine sponge *Dysidea herbacea,* and production of an antifungal iturin family, e.g., iturin A2 (**109**) (Figure 16), by coral associated microbes [167]. Coupled with other platforms, such as traditional bioassays, analytical IMS can reveal chemicals involved in host–microbial interactions occurring on surfaces. For instance, a recent study on the use of DESI-IMS coupled with traditional bioassays and comparative metabolomics revealed the spatial distribution of antifungal molecules, including phenolics and fatty acids, on the leave surface of the eelgrass *Zostera marina* [168].

The coupling of IMS techniques with orthogonal platforms such as micro- and macroscopic imaging can provide essential information to identify specific microscale structures and the true producer of ecologically important molecules. For instance, a study on the use of fluorescence microscopy and MALDI-MSI allowed for the linking of chemical diversity to either the host sponge *Smenospongia aurea* or to associated cyanobacterial symbionts. Specifically, the study revealed the biosynthetic origin of a series of previously reported peptide/polyketide smenamides, e.g., smenamide A (**110**) (Figure 16), to symbiotic cyanobacteria [169]. There has been recent research on the use of the spatial metabolomics workflow metaFISH, which integrates fluorescence in situ hybridization (FISH) microscopy with HR-atmospheric-pressure MALDI for the imaging of host–microbe symbioses and associated metabolic interactions. FISH is a well-established technique that allows for the identification of individual members within a microbial community through the use of fluorescence probes to perform hybridization to the 16S rRNA gene of the targeted microbes. However, this method poses challenges, including autofluorescence derived from host tissue and a lack of specificity of probe binding in corals and algae, which may lead to a decrease in the signal to noise ratio. The metaFISH pipeline enabled mapping on the spatial metabolome of a deep-sea mussel, *Bathymodiolus puteoserpentis*, and its unculturable symbiotic bacteria at micrometre scale of individual epithelial host cells. The analytical platform revealed a variation in metabolic phenotypes within a single symbiont 16S RNA phylotype, as well as the detection of microbial specialized metabolites, namely hopanoids, at the host–microbe interface [170]. A combination of atmospheric pressure scanning microprobe matrix-assisted laser desorption/ionisation high-resolution mass spectrometry and confocal laser scanning microscopy was used to study the interactions of the macroalga host *Ulva mutabilis* with two bacterial symbionts, *Roseovarius* sp. MS2 and *Maribacter* sp. MS6. Comparative metabolomic in situ analysis revealed the production of ectoine specifically by the bacterial symbiont *Roseovarius* sp. MS2. This was confirmed through the use of ectoine (**111**) as a metabolic marker in localization studies of *Roseovarius* sp. MS2 within the tripartite community (Figure 16) [171].

Marine invertebrate holobionts are complex systems comprising the host organisms and the associated microbes engaging in mutualistic or commensal interactions. For instance, studies have found that sponge-associated microbial symbionts play essential roles in maintaining the health of their hosts, including the provision of organic nutrients, the removal of toxic metabolic by-products, and the protection of the host from infections and oxidative stress [70]. In return, the microbial symbionts benefit by obtaining nutrients and shelter from their hosts. Metabolomic techniques linked with other complementary technologies can be used to investigate the metabolic cross talk underlying these interactions in the microenvironment. Since compounds are essentially the functional outcome of biological phenotypes, they can provide information on the functional state of biological systems. Such analytical techniques can be applied to elucidate the nature of microbial symbiosis and provide insights into how chemical interactions underpin the function of marine holobionts [169,172]. For instance, the use of non-targeted LCMS/MS and GC-MS to either whole sponge tissue or fractionated microbial cells from six different sponge species revealed several compounds, including 2-methylbutyryl-carnitine, hexanoyl-carnitine and carbohydrates, to be enriched in whole sponge tissue. These sponge-derived metabolites could be potential food sources for symbiotic microbes. On the other hand, compounds such as the antioxidant didodecyl 3,3′-thiodipropionate, the antagonistic compound docosatetraenoic acid and the immunesuppressor phenylethylamide were found to be associated with microbial cells. These microbial-associated compounds are potentially involved in either inter-microbial competitions or in the defenses for their hosts [173]. Other techniques, including microautoradiography (MAR) and secondary-ion mass spectrometry (SIMS), allow for the mapping of microbial activities and metabolite translocation at the single-cell level. MAR is able to detect microbial activities in marine invertebrates through the use of radioactive-labelled substrates, while SIMS, especially nanoSIMS, can map the directionality of the compounds in trophic interactions. In particular, nanoSIMS has been used to track compounds’ translocation in host–microbe interactions, including the uptake of ^15^N-ammonium by Symbiodiniaceae and its subsequent transfer to coral host cells [174], the localizing compounds within various compartments of the sea anemone (*Anemonia viridis*)-dinoflagellate symbioses [175], the tracking of ^13^C-labelled carbonate in the sponge *Cliona orientalis* [176] and the deciphering of the role of host cells and microbial symbionts in sponge heterotrophy by tracking different sources of dissolved organic matter (e.g., glucose, amino acids, algal-produced) and particulate organic matter in two sponge species with single-cell resolution [177].

Several integrated metabolomic-genomic-based workflows have been proposed to study metabolic interactions between host and microbial associations [178]. Currently, there is a wealth of publicly accessible online databases in the fields of genomics and natural products chemistry, several of which pair the two disciplines, including NRPminer, PoDP and MetaMiner [178]. In addition, fully automated methods, e.g., NRPquest (for non-ribosomal peptides) as well as MetaMiner and DeepRiPP (for ribosomally synthesized and post-translationally modified peptides) are available for linking mass spectra to molecular structures via the inferring of structural features predicted from genomic information with metabolomics [179]. Although the primary purpose of these proposed workflows is to accelerate novel microbial natural product discovery, they could be useful for chemical ecology research, particularly for deciphering the structure and/or function of specialized molecules produced by uncultivated microbes in marine symbiosis. A recent integrated genomic- and metabolic-based workflow was proposed by Kogawa and co-workers to isolate and analyze a single microbial cell obtained from a complex microbiome of the sponge *Theonella swinhoei* for the production of defensive compounds such as aurantosides, e.g., aurantoside A (**112**) (Figure 16) [180]. The analysis pipeline combined microfluidic encapsulation, Raman microscopy and integrated digital genomics (MERMAID) for the efficient identification of uncultivated microbial producers. This study highlights the importance of using complementary analytical techniques for the uncovering of cryptic microbial producers of diverse chemistry and their functional roles in marine holobionts.

## 4. Impacts of Climate Change and Human Activity on Marine Chemical Ecology

Coral reefs globally have been facing significant challenges from natural and anthropogenic stressors, including climate change, improper fishing practices, physical destruction and disease [181]. These unique marine ecosystems are perhaps one of the most diverse and productive environments on earth, offering habitat and food for at least 25% of marine dwellers and offering essential economic services and resources, such as coastal protection, tourism, fisheries and drug discovery. By one estimate, services and resources provided by coral reef ecosystems are worth as much as USD 375 billion per year. Moreover, having diverse terrestrial and marine ecosystems are not only essential for the maintenance of healthy natural habitats, but also for the bioprospecting of useful natural products. Any threats that impact ecosystems negatively could result in the degradation of habitats as well as the loss of available natural resources for the potential discovery of new medicines and biotechnological applications.

According to scientific estimates, by the end of the 21st century, the global mean sea surface temperature is predicted to increase by 3.2 to 5.4 °C due to emissions of greenhouse gases. This poses an immediate threat to corals, as increased sea surface temperature can cause bleaching due to the breakdown in the relationship between the corals and their symbiotic zooxanthellae. In addition, an increasing seawater temperature can translate to a significant reduction in marine biodiversity. It has been estimated that a 2 °C increase in water temperature may result in 15–40% of marine species being driven to extinction [182]. In terms of economic cost, valuation modeling estimates that a 20% marine species loss is equivalent to a market value loss of USD 112 billion–1.14 trillion, particularly in anti-cancer pharmaceuticals [183]. Furthermore, due to human-driven increased levels of carbon dioxide in the atmosphere, more CO_2_ is being dissolved into the ocean environment. The ocean’s average pH is currently slightly alkaline at about 8.1; however, as the ocean continues to absorb more CO_2_, this would lead to a more acidic condition. Based on modeling, the ocean surface pH is projected to decrease in the 8.05 to 7.75 range by the end of the 21st century. Coupled with nano/micro plastic contamination in the ocean, these combined threats jeopardize ecosystem functions and efforts in the conservation of marine biodiversity [184].

**Figure 17:**
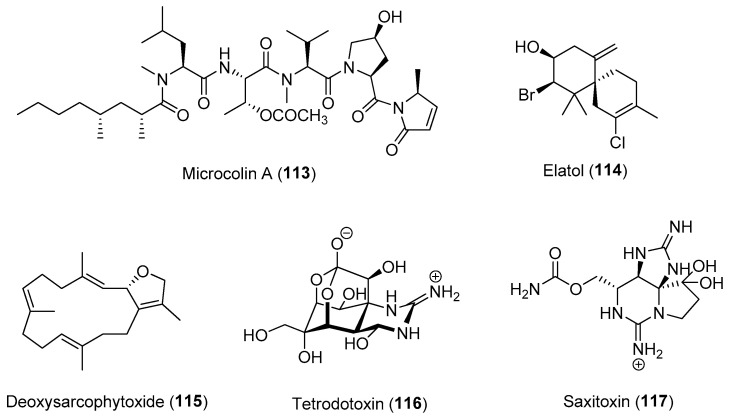
Impact of climate change on production/perception of specialized metabolites **113**–**117**.

The effects of global warming and nutrient pollution might cause the proliferation and expansion of certain taxonomic groups of marine organisms such as harmful cyanobacterial blooms at coastal areas [185,186]. Associated with the proliferation of harmful cyanobacterial blooms is the production of toxic allelochemicals, including cyanotoxins, as chemical defenses against grazers and competitors. Research has shown the combined effects of elevated temperature and the marine cyanobacterial compound microcolin A (**113**) (Figure 17) on preventing coral recruitment in the hard coral *Porites astreoides* [187]. Such simultaneous stressors could disrupt important process of coral reef recovery and resilience by negatively affecting coral survival and settlement. Climate change effects such as ocean acidification and warming could also lead to a shift of the current coral reef to either a macroalgae or sponge-dominated reef ecosystem. Studies have revealed that sponges are generally less affected by ocean warming or acidification as compared to several currently dominant benthic invertebrates, including corals. Hence, certain sponge species, particularly resilient species, are expected to benefit under near-future climate change scenarios [188].

The marine invertebrate-associated microbiome is known to provide essential ecological functions to the host macroorganisms, including nitrogen and carbon metabolism, vitamin synthesis, secondary metabolisms and host defense. Particularly in sponge species hosting photoautotrophic symbionts, these microbes can supply more than 50% of the holobiont’s energy requirements [189]. However, environmental stresses due to ocean warming, acidification, eutrophication, sedimentation and pollution can result in changes in host–microbial systems. Three possible scenarios can arise from such disturbances to the holobiont: (1) homeostasis of the holobiont by maintaining healthy baseline conditions via mechanisms of resistance or resilience, (2) an imbalance of holobiont, leading to dysbiosis and disease, and (3) acclimatization, where the holobiont reaches a new state of health in response to environmental changes [70]. In the extreme scenario, a severe disruption to the dynamic equilibrium and core diversity of the host microbiome could lead to the breakdown of the host–microbial symbiosis and ultimately the demise of the host organisms [190].

Evidence of marine microbiome changes due to environmental stressors is provided by metagenomic studies, which have revealed the alteration of core microbial communities in certain sponge species, including *Rhopaloeides odorabile*, *Xestospongia muta*, *Halichondria bowerbanki* and *Haliclona cymaeoformis* [191,192,193,194]. Such shifts in the host microbiome in response to environmental stresses can result in the loss of metabolic potentials and dysbiosis, leading to sponge disease and mortality [70,195]. There are currently gaps in our understanding of the effects of environmental perturbance on the production of secondary metabolites by microbial symbionts. However, it is not inconceivable that climate change and pollution could affect shifts in the primary/secondary metabolisms of host and microbial symbionts [196,197]. The loss of microbial symbionts due to host mortality or dysbiosis is particularly significant to natural product and drug discovery efforts since several microbial strains, such as the uncultured marine bacterial “*Candidatus* Entotheonella” and “*Ca.* Patea custodiens”, are known to produce many ecologically important bioactive molecules [198]. Although the underlying mechanisms of symbiosis are complex, the production of secondary metabolites by certain microbial symbionts in host animals can be species-specific, particularly in ascidian–symbiont associations [199]. Hence, the loss of microbial symbionts could translate to a significant loss of chemical diversity.

The impact of projected climate change on allelochemicals/secondary metabolite production has been documented in several marine macroorganisms. For examples, the effect of increased temperature causing lower levels of halogenated furanone production in the bleached thalli of the red seaweed *Delisea pulchra* has been observed [200]. These defensive halogenated furanones are known to prevent microbial pathogenic attacks by interfering with bacterial quorum-sensing systems [201]. Similarly, severe conditions of temperature (30 °C) and salinity have led to a decrease in the production of a sesquiterpene, elatol (**114**) (Figure 17), in cultivated clones of the red alga *Laurencia dendroidea* [202]. A decreased production of algal elatol could increase susceptibility of the red alga to herbivory and settlement by biofoulers [203,204]. In contrast, a marine foundation macrophyte, *Zostera marina*, was found to upregulate its chemical defense against microbial colonizers when subjected to climate extremes such as heatwaves [205].

Environmental stressors can also impair the production of defensive secondary metabolites by host organisms via the disruption to its microbial symbionts. A recent study by Farag and co-workers revealed changes in diterpenes production in soft coral *Sarcophyton* species when subjected to elevated temperature conditions [206]. Specifically, an increased temperature resulted in the photoinhibition of microbial symbionts either due to photosystem II damage or the loss of algal symbionts during coral bleaching. This led to a significant reduction in the concentrations of several diterpenes, such as sarcophytoxide (**69**) and deoxysarcophytoxide (**115**) (Figure 17), in heat stressed *S. ehrenbergi* and *S. glaucum*. The study suggested that acclimation to thermal stresses in the animal hosts is energetically costly and will cause downstream changes in the secondary metabolic pathways. A reduction in energetic contribution from the algal symbiont due to a bleaching event could result in insufficient energy reserves to maintain the high production of energetically demanding secondary metabolites. Soft coral terpenoids are known to provide chemical defenses, and the downregulation of its production due to thermal stress could increase the vulnerability of the animal to fouling and pathogenic attacks.

Functionality of secondary metabolites could be potentially affected by environmental stressors, particularly acidic conditions in the marine environment [207]. For instance, a reduced pH in the marine environment resulted in a higher proportion of protonated peptides used by a range of marine crustaceans and mollusca as signaling molecules for the coordination of brood-care and larval settlement [208]. The highly protonated peptides can cause changes in charge distribution and three-dimensional conformation from non-protonated states, which could lead to altered behavioral responses in certain crustaceans, including the green shore crab *Carcinus maenas*. Similarly, a lower pH condition led to a higher potency of two potent biotoxins, tetrodotoxin (**116**) and saxitoxin (**117**) (Figure 17), due to their protonated states [209]. These are highly neurotoxic compounds used by marine organisms such as pufferfish and dinoflagellates as defensive molecules. The enhanced potencies of these protonated neurotoxins have implications in marine ecotoxicology and have consequences for coastal ecosystem stability and services. A recent modelling study on the impact of future pH conditions on the antifouling compounds of macroalgae suggested that compounds that have phenolic and amine groups were especially sensitive to pH changes [210]. This could potentially affect the chemical defenses of certain key algal species belonging to *Phaeophyceae* and *Chlorophyta* since their antifouling compounds are highly pH sensitive. This in turn could impact the health and fitness of the host algae and could cause possible changes to the ecological responses of the macroalgal holobionts to micro and macrocolonisers. The effects on the functionality of algal compounds due to lower pH conditions could eventually lead to ecosystem restructuring and functions, such as habitat provision, offered by macroalgal hosts [210].

The perception of allelochemicals by marine organisms can also be affected by environmental changes. A recent study by Mutalipassi and co-workers provided evidence that higher CO_2_ concentrations, hence lower pH, in future marine environments will bring about significant changes in the production of volatile organic compounds by diatoms, cyanobacteria and macroalgae, found as epiphytes on the seagrass *Posidonia oceanica*, as well as in the recognition of these infochemicals perceived by marine invertebrates [211]. The behavior of several benthic and pelagic invertebrate species was also impacted when responding to volatile organic compounds produced by epiphytes, e.g., macroalgae and cyanobacteria, found on the seagrass *P. oceanica* under ocean acidification conditions. Such behavioral changes due to the pH scenario could affect the survival of key invertebrate species in the marine ecosystem [212]. Additional studies demonstrated changes to olfactory responses in other marine organisms, such as fish and invertebrates, due to lowered pH levels [207]. For instance, under low pH conditions, the larvae of the orange clownfish *Amphiprion percula* were not able to locate suitable settlement areas or distinguish between kin and non-kin neighbors [213].

## 5. Conclusions and Perspectives

The review has provided a summary of various chemical ecology-based approaches that natural product chemists can adopt in their search for novel bioactive compounds (Table 1). Despite the time-consuming nature of carrying out certain ecological investigations, such information can assist in informing drug discovery efforts in a sustainable manner. Field experiments/observations on allelopathic interactions in a specified ecosystem can lead to the selection of target marine organisms for the production of bioactive metabolites. The occurrence of the predominant taxa of benthic marine invertebrates, such as sponges, tunicates and cnidarians, could provide evidence of the presence of defensive toxic chemicals. The ecological context of allelochemicals can also be applied in bioassay-directed screening for the isolation of compounds. In addition, spatio-temporal variations in allelochemical production in marine organisms can guide optimal harvesting time for the isolation of lead compounds as well as for the detection of new analogues. As studies have shown the effects of abiotic and biotic factors on the biosynthesis of allelochemicals, these effects can be used to leverage the large-scale production of drug leads in preclinical and clinical studies.

Structurally diverse defensive allelochemicals are produced because of various species interactions engaged in competition for space and resources. The nature of these allelochemicals arising from organismal interactions can be linked to their therapeutic utility. For instance, quorum-sensing signal molecules play important ecological roles in macroalgae–bacteria interactions, which in turn can impact community organization, population structure and ecosystem functioning. Surface macroalgal-derived compounds can prevent the biofilm formation of invading marine microbial pathogens by interfering with bacterial quorum-sensing systems. In addition, several epiphytic bacteria have been reported to produce quorum-sensing interfering compounds to protect macroalgal surfaces from biofouling. Other sources of quorum-sensing modulating compounds can also be found in the phycosphere interface of phytoplankton–bacteria interactions [214]. As such, specialized metabolites derived from macroalgae/microalgae and their associated microbiomes, produced to prevent microbial pathogenic attacks, are sources of anti-biofilm and quorum-sensing inhibitory compounds [71,215]. These antimicrobial molecules, coupled with appropriate QSI bioassay, can be explored for the treatment of opportunistic pathogenic bacteria such as *Pseudomonas aeruginosa* due to their specific molecular targets of bacterial quorum-sensing systems.

An area of intense study is the chemical ecology of marine symbioses in holobiont systems. The microbial symbiotic consortium provides many essential functions, including chemical defense for the host animals. These defensive microbial-derived molecules are potential lead compounds for anticancer as well as anti-infective agents. Beneficial symbiotic microbial strains identified in the core microbiome can be isolated and cultured using conventional culturing methods to produce useful compounds. There are a number of benefits to targeting host-associated microbes, including higher chance of being cultivable than free-living microbes, and symbiont-derived molecules are ecologically ‘optimized’ to function within host systems. This is could be due to specific core microbial symbionts co-evolving with host animals, and it would benefit them to protect their hosts from pathogens and other non-commensals. Unfortunately, most microbial symbionts are largely not amenable to existing culturing techniques. As such, ecological studies should focus on the functional roles of infochemical exchanges within host–microbial or microbial–microbial interactions for the possible elicitation of natural product biosynthesis. In particular, chemical information on host-derived factors in the maintenance of host–microbe symbiosis can be harnessed for microbial fermentation and compound production. Such studies can be facilitated by recent advances/improvements in analytical techniques, including metabolomics, metagenomics, microscopy and single-cell methods [216]. Moreover, the development of a model system can provide insights into functional infochemicals that mediate host–symbiont interactions within the context of holobiont systems [217,218].

To date, there are no reports on the use of chemical ecology-based methods for the discovery of marine-derived lead compounds in either preclinical or clinical trials. The majority of these drug agents have been obtained from marine sources through random collection/screening without prior knowledge about their ecological roles. There is also a clear need to include chemical ecology research on the responses of marine holobionts to near-future climate change as this has implications for ecosystems and human health. By presenting the various chemical ecology-based strategies, coupled with innovative analytical techniques in this review, it is envisioned that ecologically relevant molecules with specific molecular targets will translate into clinical reality.

## Figures and Tables

**Table 1:** Linking of marine chemical ecology research (with examples) with drug discovery and development efforts covered in the review.

Section	Marine Chemical Ecology Research and Examples	Impact on Drug Discovery Efforts
**Section 2.1** **.**	Marine Macrobiota–Microbial Interactions	
	*Section 2.1.1*. Halogenated furanones from *Delisea pulchra.* Bromophycolides from *Callophycus serratus.*	Novel anti-infectives and cytotoxic molecules Novel molecular targets. Identity of microbial sources of bioactive compounds. Strategies for optimal culture conditions in fermentation of microbial symbionts and to elicit production of microbial compounds. Uncovering of novel biosynthetic gene clusters and its expression based on microbial genomic information. Detection of novel microbial strains for bioactive compound discovery.
	*Section 2.1.2*. Bryostatins from symbiotic γ-proteobacterial symbiont of the marine bryozoan, *Bugula neritina.* Cyclic peptides from microbial symbionts of the tunicate, *Trididemnum solidum.* Cyanobactins from cyanobacterial symbionts of the tunicate, *Prochloron didemnid.* Ecteinascidin-743 from γ-Proteobacterial symbiont of the tunicate, *Ecteinascidia turbinate.* Kahalalides from microbial symbiont of the marine alga, *Bryopsis* sp. Tambjamines from microbial symbionts of tunicates and bryozoans. Novel bioactive compounds from the microbial symbiont *Teredinibacter turnerae* T7901 of shipworms. Bioactive compounds from cyanobacterial symbiont, Hormoscilla spongeliae, of marine sponge, *Lamellodysidea herbacea.*	
	*Section 2.1.3*. Microbiome of hard corals, algae and sponges.	
**Section 2.2** **.**	Activated/Induced Chemical Defenses Activated defenses in macroalgae (e.g., *Halimeda* sp. and *Caulerpa taxifolia*), diatoms, hydroids and sponges. Induced defenses in seaweeds, sponges and phytoplanktons.	Collection strategies of marine organisms for production of more active metabolites. Use of signaling molecules for induction of bioactive compounds (e.g., anti-inflammatory agents and anti-oxidants) in marine organisms.
**Section 2.3** **.**	Allelochemicals in Competition for Space and Resources Defensive allelochemicals from a range of marine organisms, including corals, sponges, macroalgae and marine microbes.	Selection of target organisms coupled with therapeutic bioassay-directed screening. Novel anti-infectives and cytotoxic molecules. Novel molecular targets.
**Section 2.4** **.**	Allelochemicals in Phycosphere of Phytoplanktons.	Novel anti-infectives and cytotoxic molecules. Novel molecular targets. Isolation of novel microbial strains as sources of bioactive compounds. Identification of signaling molecules for expression of bioactive compounds.
**Section 2.5** **.**	Phylogeny-Based and Concerted Discovery Strategies.	New sources of marine organisms from unique ecosystem or understudied taxonomic groups for bioprospecting.
**Section 2.6** **.**	Spatial and Temporal Chemical Variation of Natural Products.	Optimal harvesting/collection of marine organisms in a sustainable manner. Increase probability of identifying new bioactive natural analogues.

## Data Availability

Not applicable.
